# Lysine Methyltransferase Inhibitors Impair H4K20me2 and 53BP1 Foci in Response to DNA Damage in Sarcomas, a Synthetic Lethality Strategy

**DOI:** 10.3389/fcell.2021.715126

**Published:** 2021-09-03

**Authors:** Ignacio Campillo-Marcos, Eva Monte-Serrano, Elena Navarro-Carrasco, Raúl García-González, Pedro A. Lazo

**Affiliations:** ^1^Molecular Mechanisms of Cancer Program, Instituto de Biología Molecular y Celular del Cáncer, Consejo Superior de Investigaciones Científicas (CSIC)-Universidad de Salamanca, Salamanca, Spain; ^2^Instituto de Investigación Biomédica de Salamanca (IBSAL), Hospital Universitario de Salamanca, Salamanca, Spain; ^3^Cancer Epigenetics Group, Josep Carreras Leukemia Research Institute (IJC), Barcelona, Spain

**Keywords:** chaetocin, tazemetostat, ionizing radiation, doxorubicin, DNA repair, histone methylation, 53BP1 foci, H2AX foci

## Abstract

**Background:**

Chromatin is dynamically remodeled to adapt to all DNA-related processes, including DNA damage responses (DDR). This adaptation requires DNA and histone epigenetic modifications, which are mediated by several types of enzymes; among them are lysine methyltransferases (KMTs).

**Methods:**

KMT inhibitors, chaetocin and tazemetostat (TZM), were used to study their role in the DDR induced by ionizing radiation or doxorubicin in two human sarcoma cells lines. The effect of these KMT inhibitors was tested by the analysis of chromatin epigenetic modifications, H4K16ac and H4K20me2. DDR was monitored by the formation of γH2AX, MDC1, NBS1 and 53BP1 foci, and the induction of apoptosis.

**Results:**

Chaetocin and tazemetostat treatments caused a significant increase of H4K16 acetylation, associated with chromatin relaxation, and increased DNA damage, detected by the labeling of free DNA-ends. These inhibitors significantly reduced H4K20 dimethylation levels in response to DNA damage and impaired the recruitment of 53BP1, but not of MDC1 and NBS1, at DNA damaged sites. This modification of epigenetic marks prevents DNA repair by the NHEJ pathway and leads to cell death.

**Conclusion:**

KMT inhibitors could function as sensitizers to DNA damage-based therapies and be used in novel synthetic lethality strategies for sarcoma treatment.

## Introduction

Chromatin structure is dynamically remodeled by DNA and histone epigenetic modifications to control and coordinate all DNA-based processes such as transcription, replication, recombination or DNA repair (Becker and Workman, 2013; Hauer and Gasser, 2017). These epigenetic modifications are mediated by several chromatin modifiers and depend on the cellular context (Ehrenhofer-Murray, 2004; Kouzarides, 2007; Kulis and Esteller, 2010; Dawson and Kouzarides, 2012). Some of these proteins that modify, or bind to, histone modifications are often dysregulated in cancer and have been used as drug targets in novel therapeutic strategies in oncology (Dawson and Kouzarides, 2012; Bates, 2020).

Histone methylation is one of the most prevalent chromatin modifications and plays a key role in the regulation, activation and silencing of genes in euchromatin, and is also associated with heterochromatin condensation (Dawson and Kouzarides, 2012). This dynamic covalent modification of histones occurs in N-terminal lysine or arginine residues (Shilatifard, 2006; DesJarlais and Tummino, 2016), and several inhibitors have been designed against lysine methyltransferases (KMTs) and demethylases (KDMs), which can be used to improve current cancer treatments (Wang and Patel, 2013; Toh et al., 2017). Among them is chaetocin, which inhibits KMTs such as SUV39H1 (Greiner et al., 2005; Chaib et al., 2012; Lai et al., 2015) or G9a (Iwasa et al., 2010; Cherblanc et al., 2013). In addition to its role as KMT inhibitor (Greiner et al., 2005), chaetocin is also a competitive inhibitor of the thioredoxin reductase, whose function is to compensate the deleterious effect of reactive oxygen species (ROS) (Tibodeau et al., 2009). Other exogenous agents such as ionizing radiation also facilitate ROS production (Jackson and Bartek, 2009; Ciccia and Elledge, 2010), causing single- and double-strand breaks (SSBs and DSBs, respectively) (Ciccia and Elledge, 2010), and their accumulation can lead to cell death (Ciccia and Elledge, 2010). Both effects contribute to chromatin relaxation and the generation of oxidative stress, which facilitate DNA damage (Isham et al., 2007; Tibodeau et al., 2009; Dixit et al., 2014; He et al., 2017). In this context, chaetocin also induces cell death in different types of tumors, including multiple myeloma (Isham et al., 2007), leukemia (Chaib et al., 2012), melanoma (Han et al., 2017), gliomas (Dixit et al., 2014; Ozyerli-Goknar et al., 2019), and gastric (Liao et al., 2019), ovarian (Li et al., 2019) and non-small cell lung cancers (Liu et al., 2015). Another recent KMT inhibitor that targets EZH2 is tazemetostat, which is clinically used in sarcomas (Italiano, 2020; Rothbart and Baylin, 2020). Furthermore, PARP1 regulates NHEJ (Non-homologous end joining) (Couto et al., 2011; Caron et al., 2019), and its inhibitors, such as olaparib, also cause DNA damage (McMahon et al., 2016) and are used in sarcomas in combination with DNA damaging agents, such as ionizing radiation (IR) (Lee et al., 2013). In turn, olaparib also sensitizes glioblastoma cells to treatment with temozolomide (Lesueur et al., 2019; Higuchi et al., 2020; Navarro-Carrasco and Lazo, 2021) and ovarian cancer cells with *BRCA* mutations (Fong et al., 2010).

SSBs and DSBs are specifically repaired by different DDR mechanisms, which are strictly coordinated in order to sequentially detect, identify, signal and repair specific DNA lesions based on their type (d’Adda di Fagagna, 2008; Jackson and Bartek, 2009). Initially, all these DDR pathways require a local distortion of chromatin caused by the DNA lesion (Ball and Yokomori, 2011; Bakkenist and Kastan, 2015), which is necessary to trigger the sequential steps in the response, ranging from chromatin remodeling and DNA protection of damaged sites, to the recognition of the type of damage and the activation of the corresponding DDR pathway (Polo, 2015; Campillo-Marcos et al., 2021). These sequential processes involve changes in covalent modifications of histones (Polo, 2015), which are necessary for the recruitment of specific DNA repair factors. In this context, histone acetylation plays an important role in response to DNA damage, since acetylated histones H3 and H4 are recognized by chromatin remodelers and protein kinases implicated in specific DDR pathways (Deem et al., 2012; Garcia-Gonzalez et al., 2020). Among them, the acetylation of histone H4 in K16 (H4K16ac) induced by DNA damage is also associated with chromatin relaxation (Murr et al., 2006; Garcia-Gonzalez et al., 2020). However, other chromatin readers depend on specific histone methylations to be recruited to DNA damage sites, such as 53BP1 (Pei et al., 2011; Wakeman et al., 2012; Zhao et al., 2020), a protein involved in non-homologous end joining (NHEJ) (Lottersberger et al., 2013; Panier and Boulton, 2014; Shibata, 2017), a key DNA repair pathway in resting cells such as neurons or cancer stem cells. In this context, the dimethylation of histone H4 in lysine 20 (H4K20me2), mediated by SET8 (Dulev et al., 2014) and NSD2/MMSET (Pei et al., 2012), is necessary for the recruitment of 53BP1 at locations with DNA damage (Botuyan et al., 2006; Pei et al., 2011; Dulev et al., 2014). H4K20me2 stabilizes the interaction between chromatin and 53BP1 in foci (Li et al., 2020), and facilitate DNA repair by the NHEJ pathway (Bunting et al., 2010; Panier and Boulton, 2014).

Due to the role of DDR in the maintenance of genome integrity and cellular homeostasis, defects in DNA repair pathways directly lead to the accumulation of SSBs and DSBs, and the subsequent cell death. In fact, patients with mutations in several DDR pathways respond much better to treatment, becoming super responders (Wheeler et al., 2021). In this work, we have studied the molecular base by which KMT inhibitors, chaetocin and tazemetostat, impair DDR (32, 52) by mimicking a DNA repair defect, which would allow their use as DNA damage sensitizers (53, 54) and become candidates for novel synthetic lethality strategies in sarcomas cells treated with either ionizing radiation (IR) (Lee et al., 2013) or doxorubicin (Maurel et al., 2009; D’Ambrosio et al., 2020).

## Materials and Methods

### Reagents and Inhibitors

Doxorubicin (Ref. 16416646, Thermo-Fisher Scientific), olaparib (AZD2281) (Ref. O-9201, LC Laboratories, Woburn, MA, United States), chaetocin (Ref. C9492, Sigma-Aldrich Merck), tazemetostat (Ref. S7128, Selleckchem) and JMJD2 inhibitor (5-carboxy-8HQ; Ref. 420201, Calbiochem, Merck-Millipore). All other reagents were from Sigma-Aldrich-Merck (Darmstadt, Germany) (Supplementary Table 1).

### Cell Lines and Culture

Two human sarcoma cell lines, U2OS (ATCC, HTB-96) from an osteosarcoma and SK-LMS-1 (ATCC, HTB-88) from a leiomyosarcoma, were validated by and obtained from the ATCC and grown as recommended by the supplier in DMEM supplemented with antibiotics, 10% FBS and 5 mM glutamine. Both cell lines are mycoplasma free. Experiments with inhibitors were performed in serum-deprived cells for 48 h to eliminate mitogenic signals.

### DNA Damage

DNA damage was induced by treatment with different doses (0.5, 1 or 3 Gy) of ionizing radiation using a Gammacell 1,000 Elite irradiator (Theratronics, Ottawa, Canada) with a ^137^Cs source. Alternatively DNA damage was also induced by treatment with doxorubicin (topoisomerase II inhibitor) or olaparib (PARP1 inhibitor) (Sanz-Garcia et al., 2012; Campillo-Marcos and Lazo, 2019). These measurements were performed in serum deprived cells (0.5%) for 48 h to remove mitogenic signaling and promote DNA repair by the NHEJ pathway. Chaetocin (100 nM) or tazemetostat (80 nM) were added at 24 h after serum withdrawal and cells were exposed to each KMT inhibitor for 24 h. When olaparib (10 μM) was combined with chaetocin, this PARP inhibitor was added 3 h later than this KMT inhibitor (27 h after deprivation of serum). In case of doxorubicin (3 μM), it was added 22 h later than chaetocin o tazemetostat (46 h after serum withdrawal), so that cells were exposed to this chemotherapeutic drug for 2 h. Finally, DNA damage caused by IR was induced after 24 h of treatment with chaetocin or tazemetostat and 48 h after deprivation of serum.

### Cell Lysates and Histone Extraction

Cells were lysed with the RIPA lysis buffer (150 mM NaCl, 1.5 mM MgCl_2_, 10 mM NaF, 4 mM EDTA, 50 mM Hepes, 1% Triton X-100, 0.1% SDS, and 10% glycerol) supplemented by phosphatases inhibitors (1 mM NaF and 1 mM sodium orthovanadate) and proteases inhibitors (1 mM PMSF, 10 μg/mL aprotinin, and 10 μg/mL leupeptin). Acidic extracts of histones were prepared as previously reported (Shechter et al., 2007). All protein extracts were quantified using the Bradford protein assay (Bio-Rad; Hercules, CA, United States). Lysates were boiled at 100°C in Laemmli buffer for 5 min for gel loading.

### Antibodies

The antibodies used are listed in Table 1 and were diluted in TBS-0.1% Tween20 or PBS-1% BSA for immunoblots and/or immunofluorescence assays, respectively.

**TABLE 1:** List of antibodies and applications.

Primary antibodies		Dilution (WB/IF)	Clone and/or reference code	Supplier
53BP1	Rabbit polyclonal	-; 1/200	H300, sc-22760	Santa Cruz Biotechnology
53BP1	Rabbit polyclonal	1/500; 1/200	NB100-304	Novus Biologicals
γH2AX	Mouse monoclonal	-; 1/200	Clone JBW301; 05-636	Millipore
MDC1	Rabbit polyclonal	1/500; 1/200	ab11169	Abcam
NBS1	Mouse monoclonal	-; 1/200	611871	BD Biosciences
NBS1 (Nibrin)	Rabbit polyclonal	1/1000; -	N 3162	Sigma-Aldrich
PARP1	Mouse monoclonal	1/1000; -	sc-8007	Santa Cruz Biotechnology
β-actin	Mouse monoclonal	1/2000; -	AC15/A5441	Sigma-Aldrich
Histone H4-K16ac	Rabbit monoclonal	1/500; 1/400	ab109463	Abcam
Histone H4-K20me2	Rabbit polyclonal	1/500; 1/100	9759	Cell Signaling
H3	Rabbit polyclonal	1/1000; -	9175	Cell Signaling
Cleaved caspase 3	Rabbit monoclonal	1/1000; -	5A1E/9664	Cell Signaling
Secondary antibodies		Dilution (WB/IF)	Reference code	Supplier
Anti-mouse IgG (WB)	Goat Anti-Mouse IgG, DyLight 680 (red)	1/10000; -	35518	Thermo Scientific
Anti-rabbit IgG (WB)	Goat Anti-Rabbit IgG, DyLight 800 (green)	1/10000; -	35571	Thermo Scientific
Goat anti-Mouse IgG (IF)	Goat anti-Mouse IgG linked to Cy3 (red)	-; 1/1000	115-165-146	Jackson ImmunoResearch
Goat anti-rabbit IgG (IF)	Goat anti-rabbit IgG linked to Cy2 (green)	-; 1/1000	111-225-144	Jackson ImmunoResearch

### SDS-Page Electrophoresis and Western Blot Analysis

Proteins were fractionated by SDS-Page vertical electrophoresis and transferred to Immobilon-FL membranes (Millipore) that were blocked with TBS-T buffer [25 mM Tris–HCl (pH 8.0), 50 mM NaCl and 2.5 mM KCl, 0.1% Tween-20] and 5% non-fat dry milk, or 5% BSA (bovine serum albumin), for 1 h at room temperature. Next, membranes were incubated with the primary antibody overnight at 4 °C, followed by three washes of 10 min in TBS-T buffer. Afterward, membranes were incubated with their corresponding secondary antibodies for 1 h in darkness, followed by three washes with TBS-T buffer (10 min). Finally, membrane signals were detected using the LI-COR Odyssey Infrared Imaging System (LI-COR Biosciences; Lincoln, NE, United States) (Sanz-Garcia et al., 2012; Monsalve et al., 2016; Campillo-Marcos and Lazo, 2019). All western blots were performed in triplicate and corresponds to the accompanying immunofluorescence figure.

### Immunofluorescence and Confocal Microscopy

Cells were plated on 60 mm dishes, which included coverslips, to be used for immunofluorescence experiments (Salzano et al., 2014, 2015; Monsalve et al., 2016; Moura et al., 2018; Campillo-Marcos and Lazo, 2019). Cells on coverslips were fixed with 3% paraformaldehyde for 30 min and treated with 200 mM glycine solution for 15 min at room temperature to eliminate the paraformaldehyde. Cells were permeabilized with 0.2% Triton X-100 solution in PBS for 30 min and blocked with 1% BSA in PBS for 30 min at room temperature or overnight at 4°C. For the simultaneous detection of two proteins, coverslips were sequentially incubated with the two primary antibodies, followed by three washes for 10 min in PBS after each one. The incubation with their corresponding secondary antibodies (Table 1), was performed for 1 h at room temperature in darkness, and finally washed three times for 10 min in PBS. Nuclei were stained with DAPI (4′, 6′-diamidino-2-phenylindole) (Sigma), diluted 1:1,000 in PBS, for 15 min at room temperature and washed three times for 10 min in PBS. Coverslips were mounted with Mowiol (Calbiochem-Merck, Darmstadt, Germany). Images were acquired with a LEICA SP5 DMI-6000B confocal microscope (Leica), with the following lasers: Argon (488 nm), DPSS (561 nm) and UV Diode (405 nm). These images were captured with a 63.0× lens zoomed in 1.5–3 × with a 1,024 × 1,024 frame and 600 Hz scanning speed and pinhole (95.6 μm), lasers intensity and photomultipliers gain and offset were maintained constant for all samples examined. Image analysis was performed with the ImageJ software^1^. These imaging experiments were independently performed three times, and one of these experiments was shown in its corresponding main or Supplementary Figure. The number of cells analyzed in each independent experiment was obtained from different fields within the same experiment and is was indicated in all box plots of each image.

### TUNEL Assays

TUNEL assay (TdT-mediated dUTP Nick-End Labeling) (Roche) was used to label free DNA-ends in damaged DNA in cells. The detection is based on the binding of fluorescein-12-dUTP to the 3′-OH of the DNA strand and was detected by a fluorescence microscope. Briefly, cells were cultured on glass coverslips and fixed with 3% PFA in PBS for 15 min at room temperature. PFA was removed and 200 mM glycine was added for 15 min. Cells were permeabilized with 0.2% triton X-100 for 15 min and blocked with 1% BSA in PBS for 30 min at room temperature or overnight at 4°C. Coverslips were incubated with 50 μl of TUNEL reaction mixture (prepared according to the manufacturer instructions) for 1 h at 37°C in darkness, followed by three washes for 10 min in PBS. Nuclei were stained with DAPI (Sigma), diluted 1:1,000 in PBS, for 10 min at room temperature, and washed three times for 10 min in PBS. Coverslips were mounted with Mowiol (Calbiochem-Merck, Darmstadt, Germany) on microscope slides. Samples were visualized using a Leica TCS SP5 DMI-6000B confocal microscope (Leica) and analyzed with the ImageJ software. As western blots and immunofluerescences, TUNEL assays were independently performed in triplicate and one representative experiment for each cell line was selected for the main figure. The number of cells in each independent experiment is indicated in the corresponding figure.

### Statistical Analysis

The IBM SPSS 25 statistics package was used for analysis. Statistical significance was calculated using Mann–Whitney *U* tests, which analyze differences between only two pairs of samples, or Dunn’s multiple comparison tests whether more than two samples were assessed at the same time, and not all of them were adjusted to a normal distribution (Bremer and Doerge, 2009). For the study we followed the ASBC recommendations (Pollard et al., 2019).

## Results

### Chaetocin Induces DNA Damage by Itself and Impairs the Recruitment of 53BP1 at Damage Sites

**FIGURE 1:**
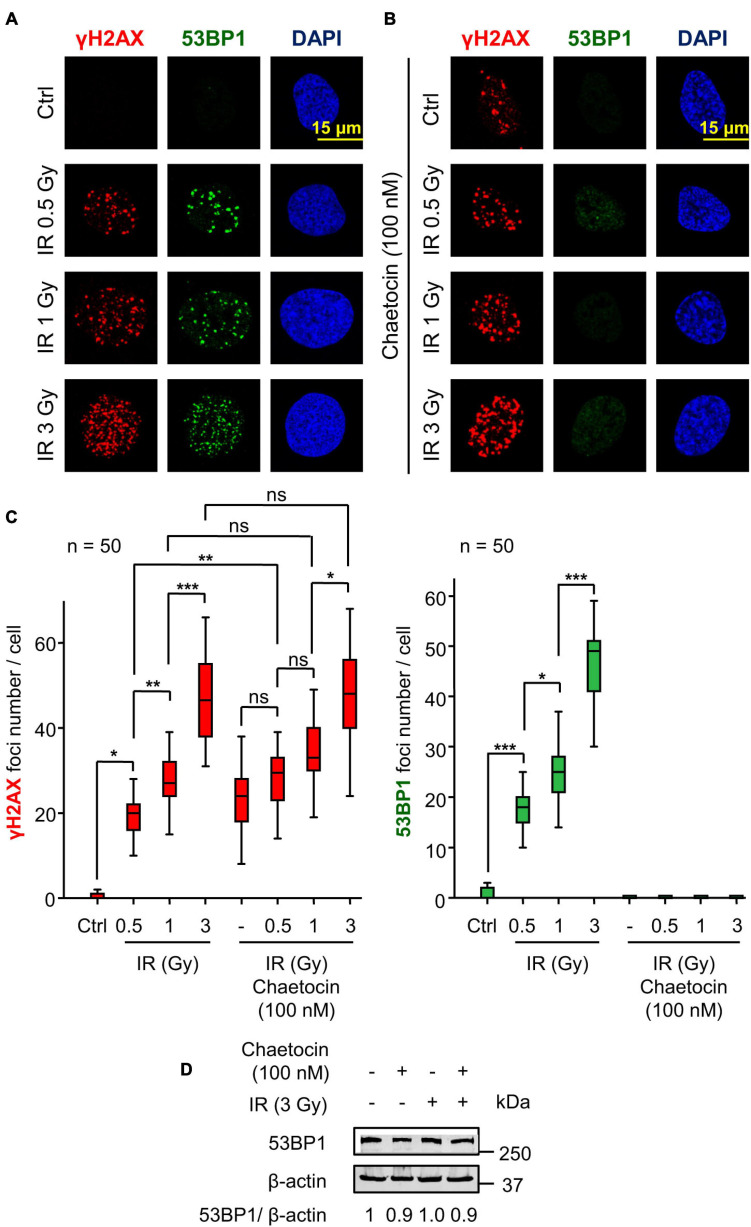
Chaetocin impairs the assembly of 53BP1 foci in response to ionizing radiation (IR) in U2OS cells deprived of serum. **(A)** γH2AX and 53BP1 foci formation in response to different doses of IR. **(B)** Effect of chaetocin on γH2AX and 53BP1 foci formation after inducing DNA damage with different doses of IR. **(C)** Quantification of γH2AX (left) and 53BP1 (right) foci in response to chaetocin and/or IR. **(D)** The immunoblot shows that chaetocin and/or IR have no effect on 53BP1 protein levels. ns, not significant. *p < 0.05, **p < 0.01, and ***p < 0.001. These images only show the detail in one cell selected for presentation. The field images are shown in Supplementary Figure 1. Ctrl, control without IR.

Initially, we tested whether chaetocin, a KMT inhibitor, was able to induce DNA damage, which was detected by the formation of γH2AX foci, a surrogate marker of DDR early steps (van Attikum and Gasser, 2005; Bekker-Jensen and Mailand, 2010; Salzano et al., 2015). Chaetocin significantly increased the number of γH2AX foci with respect to control cells (Figures 1A–C). Furthermore, the combination of this inhibitor with lower doses of IR (0.5 Gy), another source of oxidative stress, showed a synergic effect on the assembly of these foci (Figure 1C, left). However, this effect was not observed with higher doses of IR (1 or 3 Gy) in U2OS osteosarcoma cells (Figures 1A–C) and SK-LMS-1 leiomyosarcoma cells (Supplementary Figure 2).

After the initial phosphorylation of histone H2AX, additional repair proteins are sequentially recruited to DSBs. One of these proteins is 53BP1 (Pei et al., 2011; Wakeman et al., 2012), which determines DNA repair by the NHEJ pathway (Mirman and de Lange, 2020; Zhao et al., 2020). We studied whether chaetocin interfered with the assembly of 53BP1 foci in serum-deprived cells. The formation of these 53BP1 foci was impaired in U2OS osteosarcoma cells treated with chaetocin independently of the dose of IR used (Figures 1B,C). No differences in 53BP1 protein levels were detected by western blot after chaetocin and/or IR treatments (Figure 1D), which rules out a reduction of endogenous 53BP1 protein as the cause of foci loss. Similar results were obtained in SK-LMS-1 leiomyosarcoma cells (Supplementary Figure 2). Therefore, we concluded that chaetocin impairs the recruitment of 53BP1, but not of γH2AX, to DSBs induced by IR.

### The Effect of Chaetocin on 53BP1 Foci Formation Is Independent of the Agent Used to Induce DNA Damage

**FIGURE 2:**
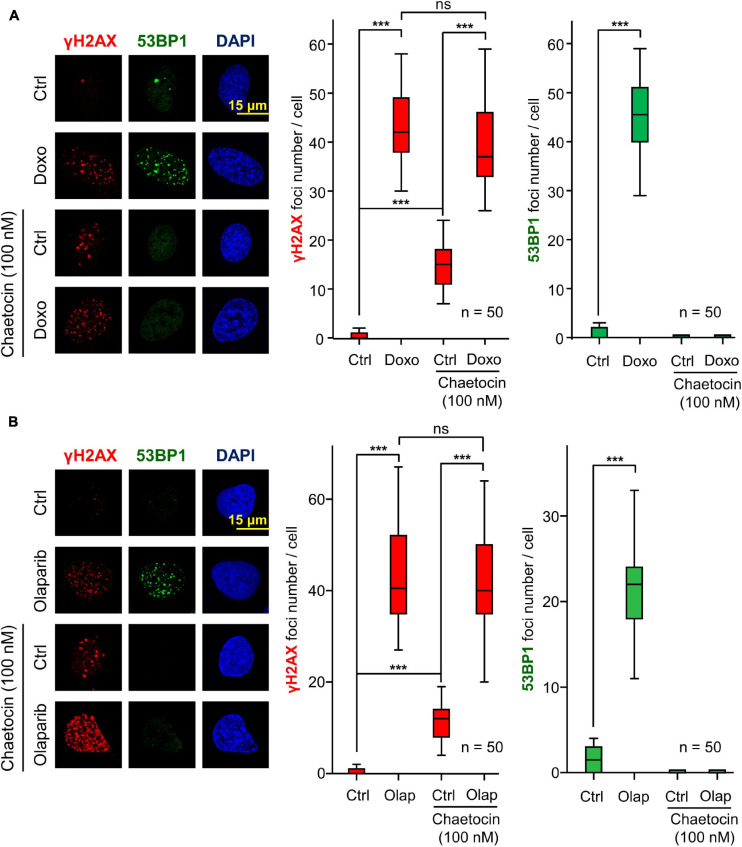
Effect of chaetocin on the formation of γH2AX and 53BP1 foci after inducing DSBs with doxorubicin (Doxo) **(A)** and olaparib (Olap) **(B)** in U2OS cells deprived of serum mitogenic signals. The effect of chaetocin on these foci in response to IR in SK-LMS-1 cells is shown in Supplementary Figure 2. Quantifications of these nuclear foci in response to chaetocin and/or doxorubicin or olaparib are shown to the right. ns, not significant, ***p < 0.001. These images only show the detail in one cell selected for presentation. The field images are shown in Supplementary Figure 3. Ctrl, control without doxorubicin or olaparib, as appropriate.

Next, we ruled out that the effect of chaetocin on the assembly of 53BP1 foci was dependent on the type of agent used to cause DNA damage. For this aim, we tested the effect of chaetocin on 53BP1 foci induced by doxorubicin, which targets topoisomerase II, or olaparib, a specific PARP1 inhibitor that is used in cancer treatment by itself or in combination with other drugs as a form of synthetic lethality (Dungey et al., 2009; Tewari et al., 2015; Ramakrishnan Geethakumari et al., 2017; Robson et al., 2017; Chabanon et al., 2019). Furthermore, olaparib is also lethal in sarcomas in combination with other agents causing DNA damage (Lee et al., 2013). Therefore, the effect of chaetocin and doxorubicin or olaparib on γH2AX and 53BP1 foci induced by these drugs was determined. The KMT inhibitor chaetocin interferes with the formation of 53BP1 foci, but did not affect γH2AX foci formation, as a result of treating cells with either doxorubicin (Figure 2A) or olaparib (Figure 2B). These results indicated that chaetocin alters the DNA damage response by impairing 53BP1 foci formation independently of the agent causing DSBs. Moreover, this effect occurs after the formation of γH2AX foci and previous to 53BP1 foci assembly.

The lack of 53BP1 foci in response to the combination of IR and chaetocin might be a consequence of a delay in the assembly of these foci caused by chaetocin, which might require longer times. Because of that, we performed a time curve after chaetocin treatment and/or IR exposure and analyzed both γH2AX and 53BP1 foci formation at different time points. We observed that 53BP1 foci did not assemble in response to chaetocin independently of the post-irradiation time, but this protein was diffusely accumulated in nuclei. However, the number and size of γH2AX residual foci (720 min post-IR) increased significantly (Supplementary Figure 4), and repair was stalled and did not progress. These results demonstrated that DDR is not working efficiently in presence of chaetocin, implying that the sequential response, which depends on 53BP1, is blocked. Consequently, earlier proteins participating in this process, such as γH2AX, are accumulated at DNA damaged sites, whose repair is stalled.

### MDC1 and NBS1, Intermediate DDR Proteins Between γH2AX and 53BP1 Foci Formation, Are Not Affected by Chaetocin Treatment

**FIGURE 3:**
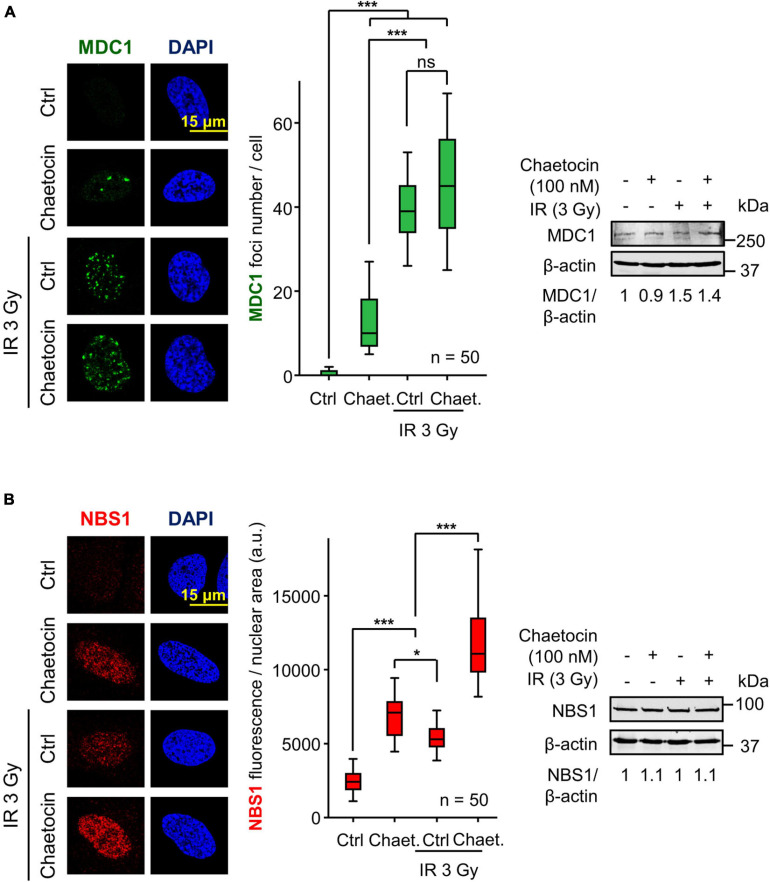
Chaetocin does not interfere with MDC1 foci formation and NBS1 accumulation after inducing DSBs by IR in U2OS cells deprived of serum. **(A)** Effect of chaetocin on MDC1 foci in response to IR (3 Gy). Quantification of MDC1 foci after treating cells with chaetocin, IR and their combination (center). The immunoblot reflects that no variations in MDC1 protein levels are induced by chaetocin and/or IR (right). ns, not significant. ***p < 0.001. **(B)** Effect of chaetocin on NBS1 accumulation in response to IR (3 Gy) in U2OS cells. Quantification of NBS1 nuclear fluorescence after treating cells with chaetocin, IR and their combination (center). The immunoblot reflects that no variations in NBS1 protein levels were induced by chaetocin and/or IR (right). ns, not significant, *p < 0.05, ***p < 0.001. Ctrl, control without IR. These images only show the detail in one cell selected for presentation. The field images are shown in Supplementary Figure 5. Ctrl, control without chaetocin. Chaet, chaetocin (100 nM).

The defective formation of 53BP1 foci might be a consequence of a disruption of previous steps in the sequential response to IR. Among them is the assembly of MDC1 and NBS1 foci, two proteins that are phosphorylated in response to IR and mediate the accumulation of 53BP1 at DSBs (Eliezer et al., 2009; Lavin et al., 2015; Monsalve et al., 2016). To determine whether chaetocin impairs the formation of MDC1 and NBS1 foci, cells were treated with chaetocin, DNA damage was induced by IR and the formation of MDC1 and NBS1 foci was studied. Chaetocin did not interfere with either MDC1 (Figure 3A) or NBS1 foci formation (Figure 3B) in response to IR, which are two sequential steps between γH2AX and 53BP1 foci in DDR, and their protein levels were not affected. This suggests that this DDR failure is due to the deficient recruitment of 53BP1 at locations with damaged DNA as a consequence of chaetocin treatment.

### H4K20me2 Induced by DNA Damage Are Impaired by Chaetocin Treatment

**FIGURE 4:**
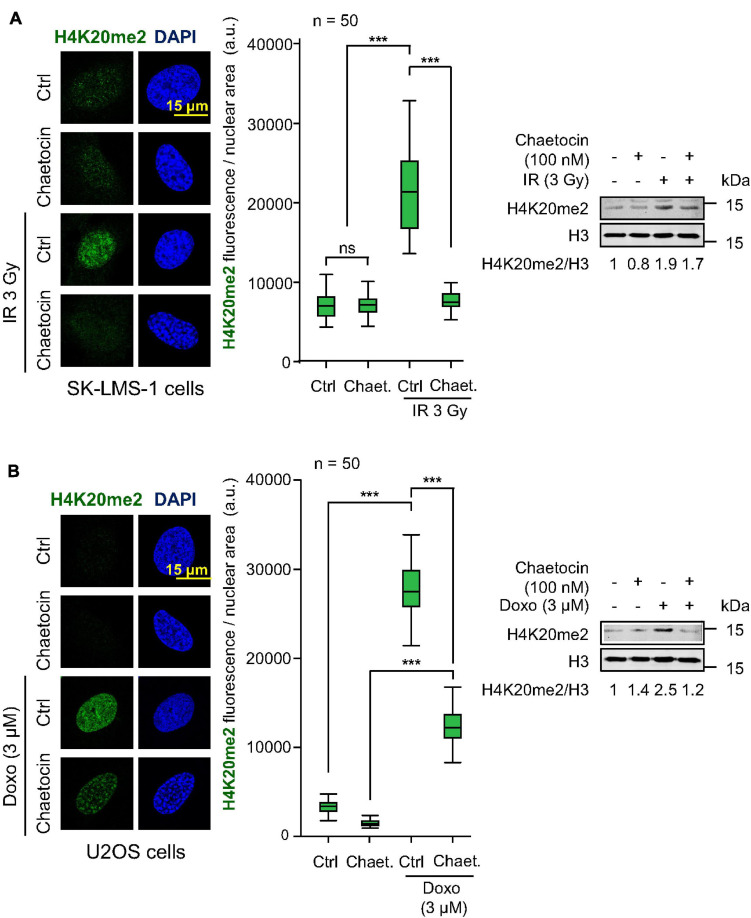
Effect of chaetocin on H4K20me2 induced by IR or doxorubicin. **(A)** Effect of chaetocin on H4K20me2 induced by IR in SK-LMS-1 cells. **(B)** Effect of chaetocin on H4K20me2 induced by doxorubicin in U2OS cells. Quantification of nuclear fluorescence associated with H4K20 dimethylation in response to chaetocin, IR, doxorubicin or their combination (center). Analysis of H4K20me2 levels by western blot (right). Ctrl, control without IR or doxorubicin, as appropriate. ns, not significant, ***p < 0.001. These images only show the detail in one cell selected for presentation. The field images are shown in Supplementary Figure 6. Ctrl, control without chaetocin. Chaet, chaetocin (100 nM).

One of the chromatin modifications that mediates the recruitment of 53BP1 at DSBs is the dimethylation of the histone H4 at lysine 20 (H4K20me2), which is directly recognized by the TUDOR domains of 53BP1 (Pei et al., 2011; Wilson et al., 2016). Therefore, we tested the ability of chaetocin to modify H4K20me2 levels in response to IR or doxorubicin in U2OS and SK-LMS-1 sarcoma cells. The treatment of both cell lines with the combination of chaetocin and IR resulted in a significant reduction of H4K20me2 levels (Figure 4A, Supplementary Figure 7A). Furthermore, this reduction in H4K20 dimethylation levels was also observed in response to doxorubicin and chaetocin treatments (Figure 4B, Supplementary Figure 7B). This effect on H4K20me2 would explain why this KMT inhibitor only affects the accumulation of 53BP1, but not of γH2AX, NBS1 or MDC1, at damaged sites.

**FIGURE 5:**
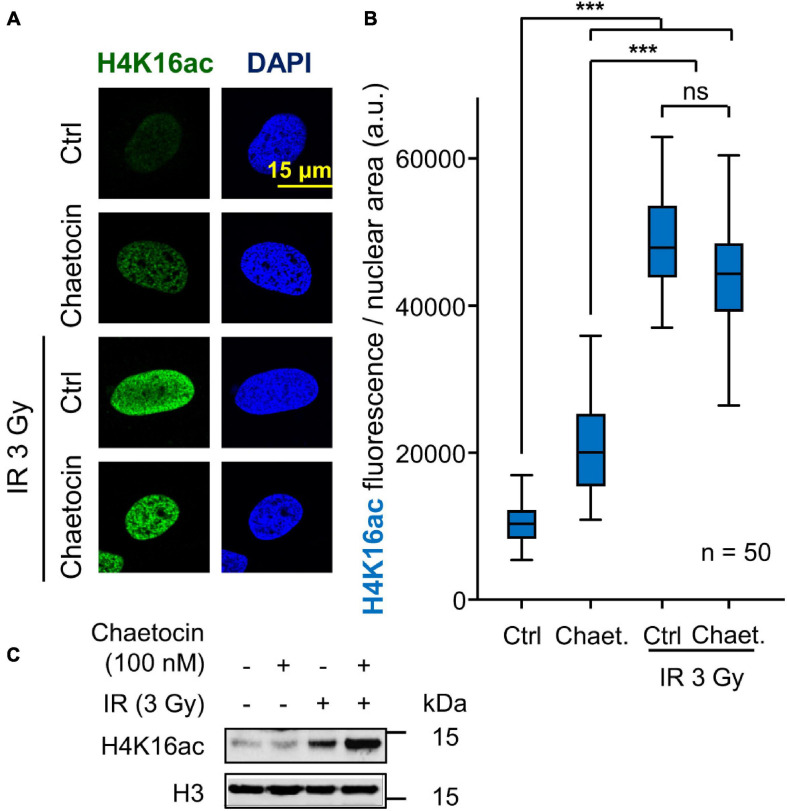
Effect of chaetocin on the acetylation of histone H4 in lysine 16 (H4K16ac) in U2OS cells treated with IR. **(A)** Detection of H4K16ac by immunofluorescence. **(B)** Quantification of the increase in H4K16ac induced by IR. ns, not significant, ***p < 0.001. **(C)** Immunoblot to show the increase of H4K16ac levels by IR. These images only show the detail in one cell selected for presentation. The field images are shown in Supplementary Figure 8. Ctrl, control without chaetocin. Chaet, chaetocin (100 nM).

DNA damage causes an early local relaxation of chromatin that is associated with the acetylation of histone H4 in K16 (H4K16ac) (Li and Wang, 2017; Garcia-Gonzalez et al., 2020). Therefore, we also determined H4K16 acetylation levels, which would have to decrease in order to enable 53BP1 to bind to chromatin through H4K20me2 (Hsiao and Mizzen, 2013; Lottersberger et al., 2013). As expected, these H4K16 acetylation levels increased after inducing DNA damage by IR and, in a smaller degree, by chaetocin, since this modification is a very early step in DDR, but such high levels remain high in response to the combination of IR and chaetocin (Figure 5). Based on these results and taking into account that chaetocin impairs 53BP1 foci formation, we suppose that IR increases H4K16ac levels in a transient manner, which facilitate the assembly of 53BP1 foci just when such levels start to decrease, whereas chaetocin, by itself or in combination with IR, not only reduces H4K20me2 levels, but also interferes with H4K16 deacetylation. Altogether, these data reinforce the idea that KMT inhibitors like chaetocin involve changes in the histone epigenetic code that interfere with DNA repair by the NHEJ pathway.

### Tazemetostat Impairs H4K20me2 the DNA Damage Response Induced by Doxorubicin

**FIGURE 6:**
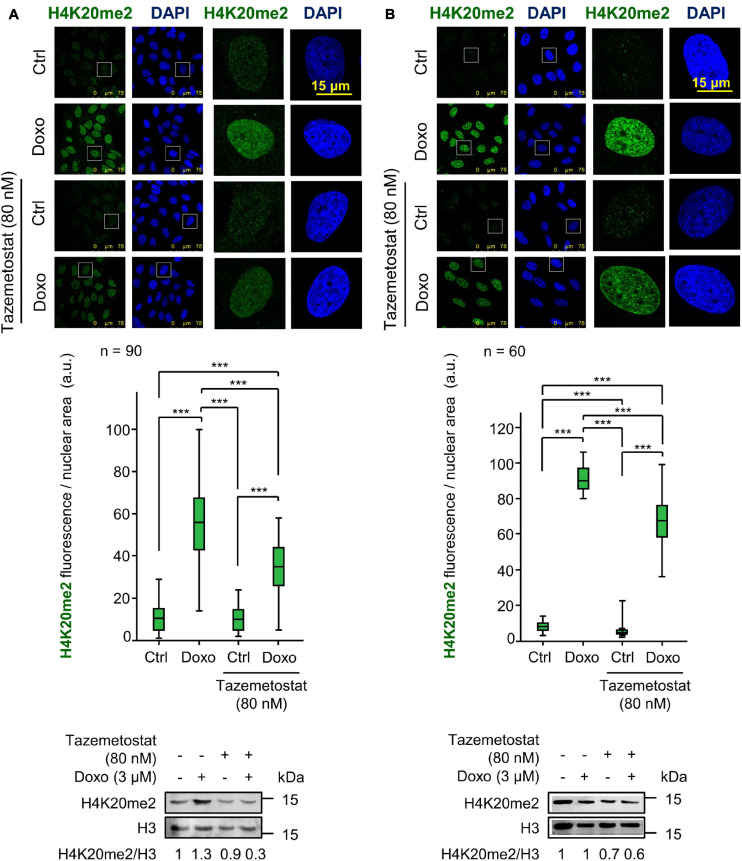
Effect of tazemetostat (TZM) on H4K20me2 levels in response to DNA damage induced by doxorubicin. **(A)** Effect of tazemetostat on the levels of H4K20me2 in U2OS cells deprived of serum. The tazemetostat effect on γH2AX and 53BP1 is shown in Figure 7. **(B)** Effect of tazemetostat on the levels of H4K20me2 in SK-LMS-1 cells. The tazemetostat effect on γH2AX and 53BP1 is shown in Supplementary Figure 9. ***p < 0.001. Ctrl, control without doxorubicin.

**FIGURE 7:**
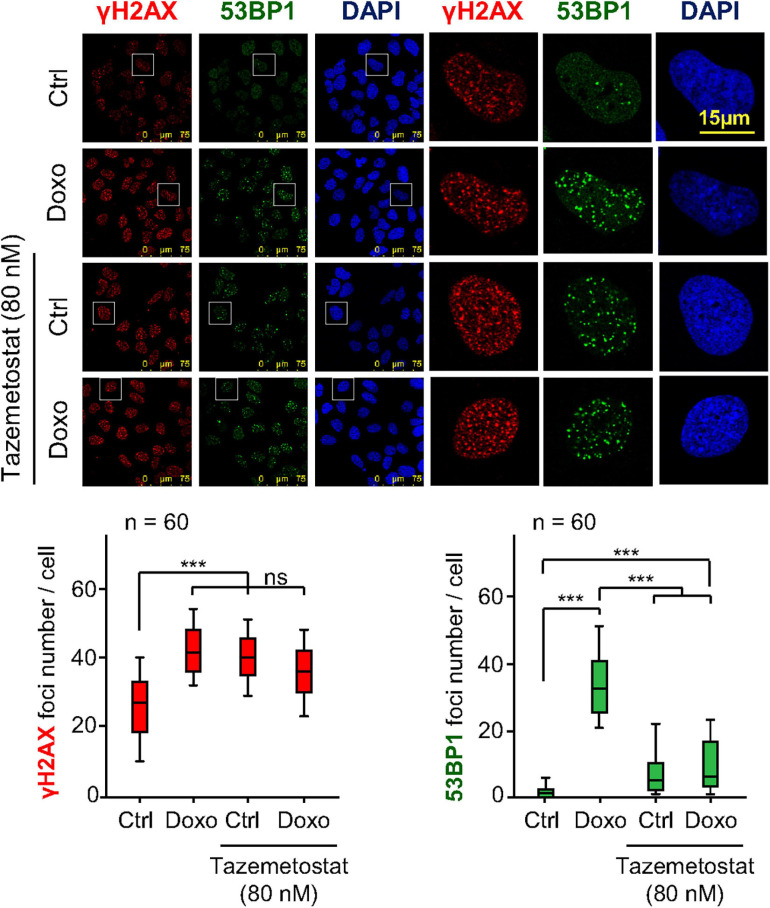
Effect of tazemetostat on γH2AX and 53BP1 foci in response to doxorubicin treatment in U2OS osteosarcoma cells deprived of serum. Effect of tazemetostat on the formation of γH2AX (red) and 53BP1 (green) foci. The detail images selected indicated by boxes are shown to the right. Graphs at the bottom show the quantification of γH2AX and 53BP1 foci. ns, not significant. ***p < 0.001. Ctrl, control without doxorubicin.

Tazemetostat is a KMT inhibitor targeting EZH2 (Italiano et al., 2018) and G9a (Soumyanarayanan and Dymock, 2016; Dockerill et al., 2020) that has been approved for the treatment of sarcomas (Italiano et al., 2018; Gounder et al., 2020) and sensitizes ovarian cells to DNA damage (Karakashev et al., 2020). Therefore, we tested whether tazemetostat could also impair the H4K20me2 as part of the DDR in sarcoma cells treated with doxorubicin. The treatment with tazemetostat significantly reduced the levels of H4K20me2 in response to doxorubicin in U2OS (Figure 6A) and SK-LMS-1 (Figure 6B) cells as well as inhibited the formation of 53BP1 foci induced by doxorubicin in U2OS cells (Figure 7) and SK-LMS-1 cells (Supplementary Figure 9).

### JMJD2 Inhibitor Does Not Alter the Formation of 53BP1 Foci Induced by DNA Damage

**FIGURE 8:**
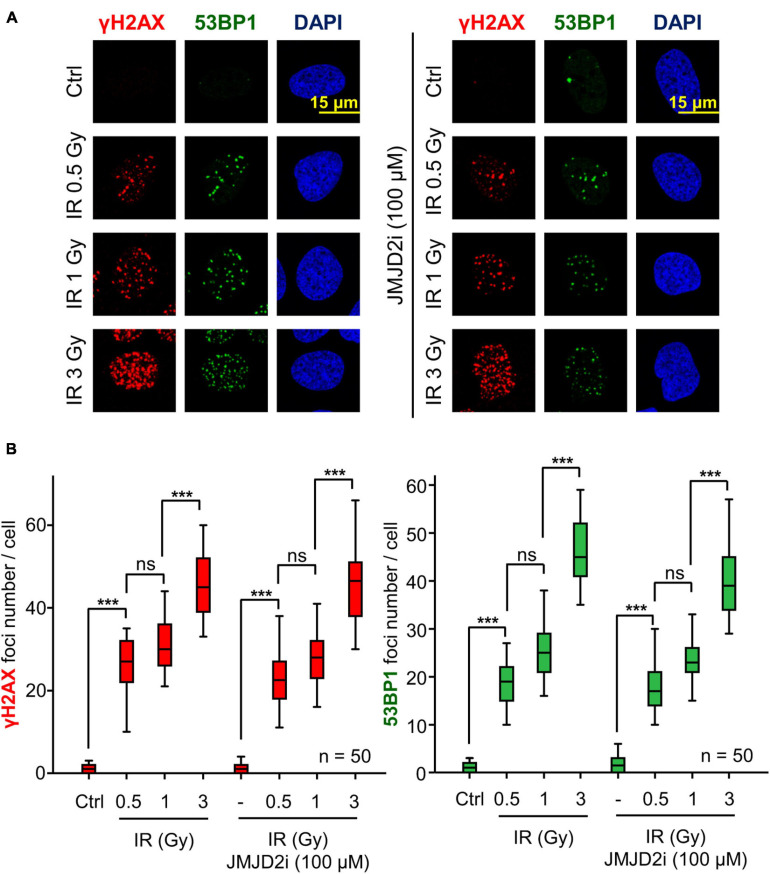
The assembly of 53BP1 foci is not affected by JMJD2i treatment, which inhibits lysine demethylation, after inducing DSBs with IR in U2OS cells. **(A)** γH2AX and 53BP1 foci formation in response to JMJD2 inhibitor and/or IR. **(B)** Quantification of γH2AX and 53BP1 foci after JMJD2i treatment and inducing DNA damage by IR. ns, not significant, ***p < 0.001. The field images are shown in Supplementary Figure 10. Ctrl, control without IR.

53BP1 is an essential mediator protein in NHEJ pathway and its recruitment at damage sites depends on covalent modifications in specific histones residues and repair proteins. Since chaetocin or tazemetostat, KMT inhibitors, impair the accumulation of 53BP1 at damage sites in response to DNA damage, we studied the consequences of treatment with a lysine demethylase (KDM) inhibitor, JMJD2i, on 53BP1 foci formation. Our results showed that these foci were correctly formed after the combination of JMJD2i and different doses of IR (Figure 8). This result supports our hypothesis that the effect of chaetocin and tazemetostat on 53BP1 foci formation is modulated by variations of histone methylation patterns, since high methylation levels associated with the treatment with JMJD2i facilitates the recruitment of 53BP1 in response to DNA damage.

### Chaetocin and Tazemetostat Facilitate DNA Damage Induced by Doxorubicin

**FIGURE 9:**
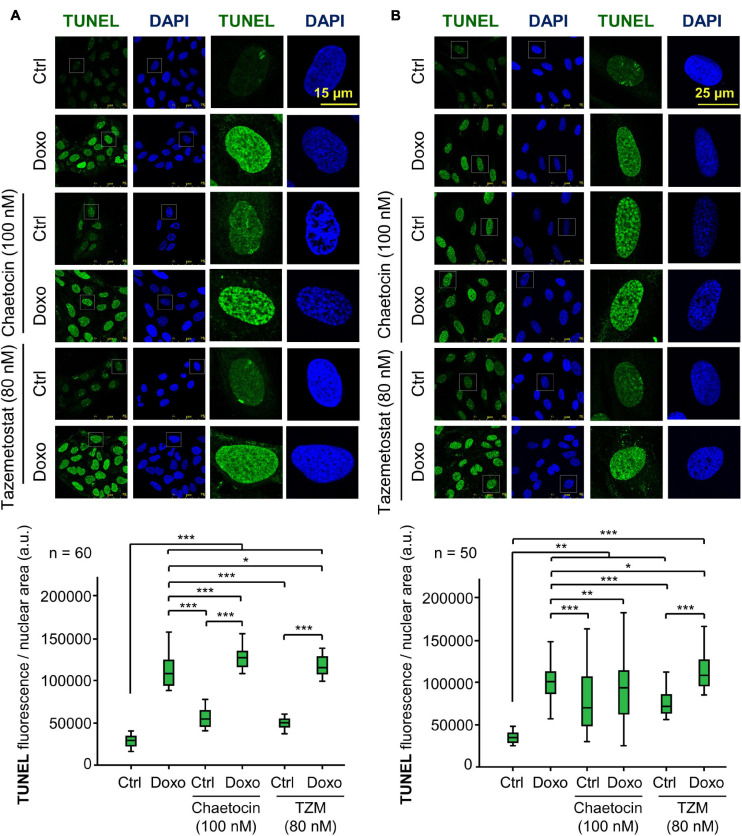
Effect of chaetocin and tazemetostat on the induction of DNA damage detected in TUNEL assays in U2OS **(A)** and SK-LMS-1 **(B)** sarcoma cells deprived of serum. DNA damage was detected by labeling free DNA ends. *p < 0.05, **p < 0.01, and ***p < 0.001. Ctrl, control without doxorubicin.

A consequence of KMT inhibition would be a facilitation of chromatin relaxation, which makes tumor cells more susceptible to undergo DNA damage. Therefore, we tested whether these two KMT inhibitors, chaetocin and tazemetostat, could really cause DNA damage by themselves or in cooperation with doxorubicin. The accumulation of DNA damage was detected by labeling nuclear free-DNA ends in a TUNEL assay. These two KMT inhibitors by themselves caused a minor, but significant increase in DNA damage, which reached their maximum effect when they were combined with doxorubicin in both U2OS (Figure 9A) and SK-LMS-1 cells (Figure 9B).

### Chaetocin and Tazemetostat Promote Cell Death in Response to DNA Damage Induction

**FIGURE 10:**
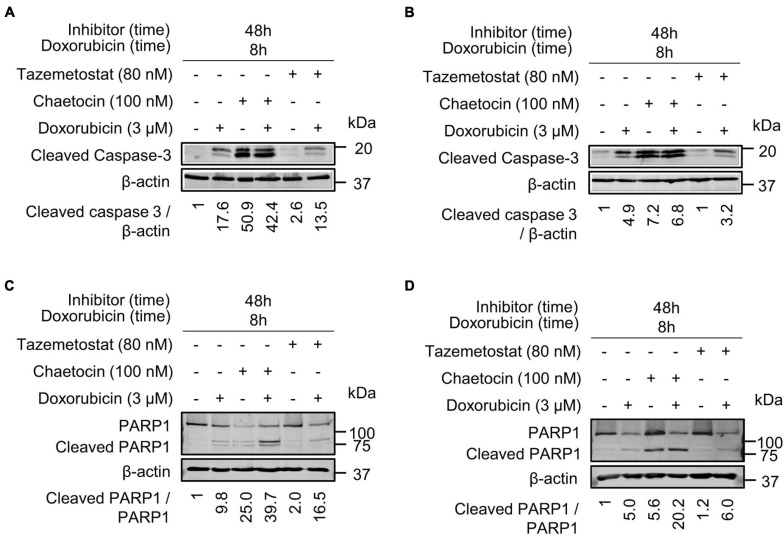
Chaetocin and tazemetostat cause an activation of caspases detected by the processed caspase 3 and PARP1 in doxorubicin treated cells. Effect of chaetocin and tazemetostat on processed caspase 3 after treatment with doxorubicin in U2OS **(A)** and SK-LMS-1 **(B)** cells deprived of serum. Effect of chaetocin and tazemetostat on PARP1 cleavage after treatment with doxorubicin in U2OS **(C)** and SK-LMS-1 **(D)** cells deprived of serum. The cleavage of PARP1 in response to chaetocin is shown in Supplementary Figure 11.

Next, we analyzed the effect of these inhibitors, chaetocin and tazemetostat, on the induction of apoptosis by detection of the cleavage of caspase 3 and PARP1. PARP1 is cleaved by activated caspase 3 and can be detected as a smaller protein with a specific antibody for this cleaved fragment. Because of that, the cleavage of caspase-3 and PARP1 was determined in both sarcoma cell lines. Cleaved caspase 3 was detected after chaetocin or tazemetostat treatments either by themselves or in combination with doxorubicin at different time points in both cell lines (Figures 10A,B). Chaetocin by itself was able to induce caspase activation at shorter times than tazemetostat, whose effect required a longer time and the cooperation of doxorubicin. The processing of PARP1 as a result of caspase activation was similarly determined (Figures 10C,D). Chaetocin was also more effective at a shorter time than tazemetostat and PARP1 processing that was induced by these drugs in cooperation with doxorubicin. This effect of chaetocin on PARP1 processing induced by ionizing radiation was further confirmed in U2OS and SK-LMS-1 cell lines (Supplementary Figure 11).

## Discussion

Genomic instability contributes to cancer development. However, the generation of excessive DNA damage by treatment combinations can lead to a loss of tumor cell viability. In this context, approaches promoting DNA damage accumulation in tumor cells can be exploited in novel cancer therapeutic strategies of synthetic lethality. Among potential targets are those proteins involved in different DDR pathways either by combining them in tumors with mutations in a DDR gene, such as *BRCA1* (Fong et al., 2010) or *WRN* (Chan et al., 2019; Kategaya et al., 2019; Lieb et al., 2019), or by pharmacological targeting of another DDR pathway, such as olaparib, which targets PARP1 (Tewari et al., 2015; Leichman et al., 2016; Srinivasan et al., 2017). An alternative is to simultaneously target two different DDR pathways or associated processes, such as chromatin remodeling, with drugs to promote tumor cell death. Based on that, KMTs are good candidates, since they regulate chromatin compaction. If these KMT inhibitors were combined with DNA-based treatments, such as IR or doxorubicin, which are commonly used in sarcomas, the impact on DNA damage could be significantly higher and thus lethal for the tumor cell.

The recruitment of 53BP1 to chromatin in response to DSBs depends on two major components. One is the balance of epigenetic modifications such as H4K16ac (Murr et al., 2006; Garcia-Gonzalez et al., 2020), H2AK15ub (Fradet-Turcotte et al., 2013) and H4K20me2 (Jacquet et al., 2016; Wilson et al., 2016). The other is the sequential accumulation and phosphorylation of histone γH2AX, MDC1, and NBS1 at DNA damaged locations (Lou et al., 2006; Eliezer et al., 2009; Liu et al., 2012; Lavin et al., 2015; Monsalve et al., 2016). In this context, we have demonstrated that the inhibition of KMTs interfered with DDR and the NHEJ pathway, which require the spatial and temporal coordination of different DNA repair factors and a local dynamic remodeling of chromatin. Furthermore, the manipulation of epigenetic modifications mediated by the KMT inhibitors chaetocin and tazemetostat did not alter earlier steps in DDR, such as γH2AX, MDC1 and NBS1 foci, suggesting that KMT inhibitors work at later steps in DDR.

The dimethylation of histone H4 in lysine 20 (H4K20me2) stabilizes the interaction between chromatin and 53BP1 in foci (Li et al., 2020). Our study shows that chaetocin and tazemetostat strongly reduce H4K20me2 levels and consequently interfere with DDR due to the impairment of 53BP1 recruitment to damage locations. Chaetocin is more efficient than tazemetostat in impairing H4K20me2, which is necessary for DDR by the NHEJ pathway. This effectiveness of chaetocin is likely to be a consequence of its less specific inhibition of KMTs, while tazemetostat has a more specific target, EZH2 (Brach et al., 2017; Italiano et al., 2018; Rothbart and Baylin, 2020). Furthermore, the acetylation of H4K16 has to be removed in order to facilitate the dimethylation of H4K20 after the generation of DNA damage (Hsiao and Mizzen, 2013; Tang et al., 2013). However, H4K16ac levels were still high in response to IR and chaetocin, making more difficult that H4K20 can be methylated and impairing 53BP1 recruitment to DNA damage locations. Apart from the modulation of these epigenetic modifications, the disruption of H2AK15 ubiquitination and its writer, the RNF168 ubiquitin ligase, could also indirectly contribute to the impairment of 53BP1 foci formation. However, further studies should be performed in order to confirm this hypothesis.

Along with their effect on H4K20me2, both chaetocin and tazemetostat cause DNA damage by themselves, but this effect is particularly higher in the case of chaetocin. This effect is a likely consequence of the generation of oxidative stress by chaetocin, which is a competitive substrate, and inhibitor, of the thioredoxin reductase (Tibodeau et al., 2009) leading to an increase of reactive oxygen species (ROS), which are directly related to DNA damage and promote cell death (Bruner et al., 2000; Hoeijmakers, 2001; van Gent et al., 2001; Isham et al., 2007; Chaib et al., 2012; Han et al., 2017; He et al., 2017; Li et al., 2019; Ozyerli-Goknar et al., 2019; Wen et al., 2019). This effect of chaetocin on ROS production and its lower KMT specificity make it more toxic than tazemetostat.

Due to the DDR impairment caused by KMT inhibitors, chaetocin or tazemetostat, DNA damage is accumulated and leads to tumor death. The treatment with these two KMT inhibitors caused an increase in DNA damage, higher when cells were treated with IR or doxorubicin, which was detected by labelling of free DNA-ends in TUNEL assays, and an increase in processed PARP1 and caspase-3, which indicates that apoptosis has been activated in tumor cells. All these results are consistent with the role of these KMT inhibitors in facilitating cell death (Duriez and Shah, 1997; Soldani and Scovassi, 2002).

**FIGURE 11:**
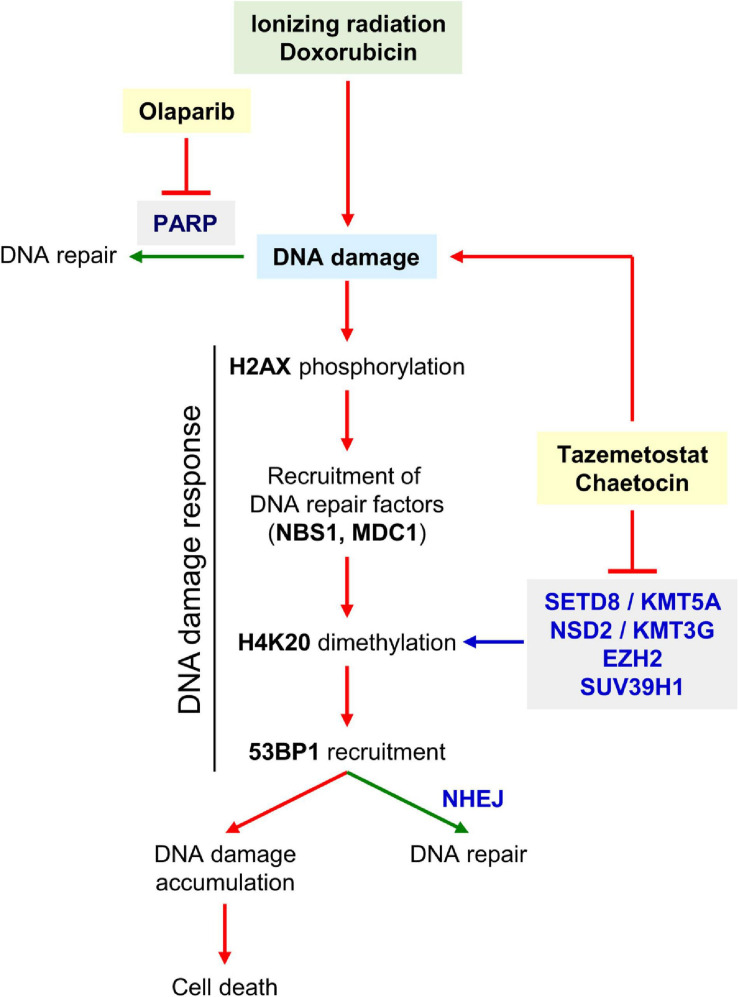
Diagram showing the induction of DNA damage by radiation or doxorubicin, and the effect of KMT inhibitors on DDR. Both inhibitors promote the generation of DNA damage, impair its repair and thus contribute to facilitate tumor cell death. The SUV39H1, EZH2, SETD8, and NSD2, which mediate H4 lysine methylation, would be the most probable candidate enzymes inhibited by chaetocin and/or tazemetostat and explain why H4K20me2 levels decrease in presence of both KMT inhibitors.

Therefore, the combination of a KMT inhibitor with current treatments based on DNA damage in sarcomas, such as ionizing radiation or doxorubicin, could be useful to develop novel strategies that sensitize tumor cells (Figure 11) and, at the same time, might contribute to reduce the dose of these toxic treatments. The consequence would be a reduction of treatment side effects that would lead to an improvement in patient quality of life and life expectancy.

## Conclusion

The inhibition of KMTs by chaetocin or tazemetostat causes a significant reduction of H4K20me2 levels, which impairs the recruitment and formation of 53BP1 foci induced by DNA damage. As a consequence of this defective DDR, there is an accumulation of DNA damage that promotes tumor cell death. Based on these effects, we propose that new therapeutic approaches against tumor cells could be based on the use of these KMT inhibitors in synthetic lethality strategies to treat cancer.

## Data Availability Statement

The original contributions presented in the study are included in the article/Supplementary Material, further inquiries can be directed to the corresponding author/s.

## Author Contributions

IC-M performed the experiments, analyzed the data, and wrote the manuscript. RG-G, EN-C, and EM-S performed the experiments. PL designed and coordinated this work, analyzed the data, and wrote the manuscript. All authors contributed to the article and approved the submitted version.

## Conflict of Interest

The authors declare that the research was conducted in the absence of any commercial or financial relationships that could be construed as a potential conflict of interest.

## Publisher’s Note

All claims expressed in this article are solely those of the authors and do not necessarily represent those of their affiliated organizations, or those of the publisher, the editors and the reviewers. Any product that may be evaluated in this article, or claim that may be made by its manufacturer, is not guaranteed or endorsed by the publisher.

## Footnotes

1
https://imagej.nih.gov/ij/

## References

[B1] Bakkenist C. J. Kastan M. B. (2015). Chromatin perturbations during the DNA damage response in higher eukaryotes. *DNA Repair* 36 8–12. 10.1016/j.dnarep.2015.09.002 26391293 PMC4727245

[B2] Ball A. R. Jr. Yokomori K. (2011). Damage site chromatin: open or closed? *Curr. Opin. Cell Biol.* 23 277–283. 10.1016/j.ceb.2011.03.012 21489773 PMC3109140

[B3] Bates S. E. (2020). Epigenetic therapies for cancer. *N. Engl. J. Med.* 383 650–663. 10.1056/NEJMra1805035 32786190

[B4] Becker P. B. Workman J. L. (2013). Nucleosome remodeling and epigenetics. *Cold Spring Harb. Perspect. Biol.* 5:a017905. 10.1101/cshperspect.a017905 24003213 PMC3753709

[B5] Bekker-Jensen S. Mailand N. (2010). Assembly and function of DNA double-strand break repair foci in mammalian cells. *DNA Repair* 9 1219–1228. 10.1016/j.dnarep.2010.09.010 21035408

[B6] Botuyan M. V. Lee J. Ward I. M. Kim J. E. Thompson J. R. Chen J. (2006). Structural basis for the methylation state-specific recognition of histone H4-K20 by 53BP1 and Crb2 in DNA repair. *Cell* 127 1361–1373. 10.1016/j.cell.2006.10.043 17190600 PMC1804291

[B7] Brach D. Johnston-Blackwell D. Drew A. Lingaraj T. Motwani V. Warholic N. M. (2017). EZH2 inhibition by tazemetostat results in altered dependency on B-cell activation signaling in DLBCL. *Mol. Cancer Ther.* 16 2586–2597. 10.1158/1535-7163.MCT-16-0840 28835384

[B8] Bremer M. Doerge R. M. (2009). *Statistics at the Bench: A Step-By Step Handbook for Biologists.* New York: Cold Spring Harbor Laboratory Press.

[B9] Bruner S. D. Norman D. P. Verdine G. L. (2000). Structural basis for recognition and repair of the endogenous mutagen 8-oxoguanine in DNA. *Nature* 403 859–866. 10.1038/35002510 10706276

[B10] Bunting S. F. Callen E. Wong N. Chen H. T. Polato F. Gunn A. (2010). 53BP1 inhibits homologous recombination in Brca1-deficient cells by blocking resection of DNA breaks. *Cell* 141 243–254. 10.1016/j.cell.2010.03.012 20362325 PMC2857570

[B11] Campillo-Marcos I. García-González R. Navarro-Carrasco E. Lazo P. A. (2021). The human VRK1 chromatin kinase in cancer biology. *Cancer Lett.* 503 117–128. 10.1016/j.canlet.2020.12.032 33516791

[B12] Campillo-Marcos I. Lazo P. A. (2019). Olaparib and ionizing radiation trigger a cooperative DNA-damage repair response that is impaired by depletion of the VRK1 chromatin kinase. *J. Exp. Clin. Cancer Res.* 38:203. 10.1186/s13046-019-1204-1 31101118 PMC6525392

[B13] Caron M. C. Sharma A. K. O’Sullivan J. Myler L. R. Ferreira M. T. Rodrigue A. (2019). Poly(ADP-ribose) polymerase-1 antagonizes DNA resection at double-strand breaks. *Nat. Commun.* 10:2954. 10.1038/s41467-019-10741-9 31273204 PMC6609622

[B14] Chabanon R. M. Muirhead G. Krastev D. B. Adam J. Morel D. Garrido M. (2019). PARP inhibition enhances tumor cell-intrinsic immunity in ERCC1-deficient non-small cell lung cancer. *J. Clin. Invest.* 129 1211–1228. 10.1172/JCI123319 30589644 PMC6391116

[B15] Chaib H. Nebbioso A. Prebet T. Castellano R. Garbit S. Restouin A. (2012). Anti-leukemia activity of chaetocin via death receptor-dependent apoptosis and dual modulation of the histone methyl-transferase SUV39H1. *Leukemia* 26 662–674. 10.1038/leu.2011.271 21979880

[B16] Chan E. M. Shibue T. McFarland J. M. Gaeta B. Ghandi M. Dumont N. (2019). WRN helicase is a synthetic lethal target in microsatellite unstable cancers. *Nature* 568 551–556. 10.1038/s41586-019-1102-x 30971823 PMC6580861

[B17] Cherblanc F. L. Chapman K. L. Brown R. Fuchter M. J. (2013). Chaetocin is a nonspecific inhibitor of histone lysine methyltransferases. *Nat. Chem. Biol.* 9 136–137. 10.1038/nchembio.1187 23416387

[B18] Ciccia A. Elledge S. J. (2010). The DNA damage response: making it safe to play with knives. *Mol. Cell* 40 179–204. 10.1016/j.molcel.2010.09.019 20965415 PMC2988877

[B19] Couto C. A. Wang H. Y. Green J. C. Kiely R. Siddaway R. Borer C. (2011). PARP regulates nonhomologous end joining through retention of Ku at double-strand breaks. *J. Cell Biol.* 194 367–375. 10.1083/jcb.201012132 21807880 PMC3153639

[B20] d’Adda di Fagagna F. (2008). Living on a break: cellular senescence as a DNA-damage response. *Nat. Rev. Cancer* 8 512–522. 18574463 10.1038/nrc2440

[B21] D’Ambrosio L. Touati N. Blay J. Y. Grignani G. Flippot R. Czarnecka A. M. (2020). Doxorubicin plus dacarbazine, doxorubicin plus ifosfamide, or doxorubicin alone as a first-line treatment for advanced leiomyosarcoma: a propensity score matching analysis from the European Organization for Research and Treatment of Cancer Soft Tissue and Bone Sarcoma Group. *Cancer* 126 2637–2647. 10.1002/cncr.32795 32129883

[B22] Dawson M. A. Kouzarides T. (2012). Cancer epigenetics: from mechanism to therapy. *Cell* 150 12–27. 10.1016/j.cell.2012.06.013 22770212

[B23] Deem A. K. Li X. Tyler J. K. (2012). Epigenetic regulation of genomic integrity. *Chromosoma* 121 131–151. 10.1007/s00412-011-0358-1 22249206 PMC3982914

[B24] DesJarlais R. Tummino P. J. (2016). Role of histone-modifying enzymes and their complexes in regulation of chromatin biology. *Biochemistry* 55 1584–1599. 10.1021/acs.biochem.5b01210 26745824

[B25] Dixit D. Ghildiyal R. Anto N. P. Sen E. (2014). Chaetocin-induced ROS-mediated apoptosis involves ATM-YAP1 axis and JNK-dependent inhibition of glucose metabolism. *Cell Death Dis.* 5:e1212. 10.1038/cddis.2014.179 24810048 PMC4047915

[B26] Dockerill M. Gregson C. Dh O. D. (2020). Targeting PRC2 for the treatment of cancer: an updated patent review (2016 - 2020). *Expert Opin. Ther. Pat.* 31 119–135. 10.1080/13543776.2021.1841167 33103538

[B27] Dulev S. Tkach J. Lin S. Batada N. N. (2014). SET8 methyltransferase activity during the DNA double-strand break response is required for recruitment of 53BP1. *EMBO Rep.* 15 1163–1174. 10.15252/embr.201439434 25252681 PMC4253490

[B28] Dungey F. A. Caldecott K. W. Chalmers A. J. (2009). Enhanced radiosensitization of human glioma cells by combining inhibition of poly(ADP-ribose) polymerase with inhibition of heat shock protein 90. *Mol. Cancer Ther.* 8 2243–2254. 10.1158/1535-7163.MCT-09-0201 19671736 PMC2728724

[B29] Duriez P. J. Shah G. M. (1997). Cleavage of poly(ADP-ribose) polymerase: a sensitive parameter to study cell death. *Biochem. Cell Biol.* 75 337–349. 9493956

[B30] Ehrenhofer-Murray A. E. (2004). Chromatin dynamics at DNA replication, transcription and repair. *Eur. J. Biochem.* 271 2335–2349. 10.1111/j.1432-1033.2004.04162.x 15182349

[B31] Eliezer Y. Argaman L. Rhie A. Doherty A. J. Goldberg M. (2009). The direct interaction between 53BP1 and MDC1 is required for the recruitment of 53BP1 to sites of damage. *J. Biol. Chem.* 284 426–435. 10.1074/jbc.M807375200 18986980

[B32] Fong P. C. Yap T. A. Boss D. S. Carden C. P. Mergui-Roelvink M. Gourley C. (2010). Poly(ADP)-ribose polymerase inhibition: frequent durable responses in BRCA carrier ovarian cancer correlating with platinum-free interval. *J. Clin. Oncol.* 28 2512–2519. 10.1200/JCO.2009.26.9589 20406929

[B33] Fradet-Turcotte A. Canny M. D. Escribano-Díaz C. Orthwein A. Leung C. C. Huang H. (2013). 53BP1 is a reader of the DNA-damage-induced H2A Lys 15 ubiquitin mark. *Nature* 499 50–54. 10.1038/nature12318 23760478 PMC3955401

[B34] Garcia-Gonzalez R. Morejon-Garcia P. Campillo-Marcos I. Salzano M. Lazo P. A. (2020). VRK1 phosphorylates Tip60/KAT5 and is required for H4K16 acetylation in response to DNA damage. *Cancers* 12:2986. 10.3390/cancers12102986 33076429 PMC7650776

[B35] Gounder M. Schoffski P. Jones R. L. Agulnik M. Cote G. M. Villalobos V. M. (2020). Tazemetostat in advanced epithelioid sarcoma with loss of INI1/SMARCB1: an international, open-label, phase 2 basket study. *Lancet Oncol.* 21 1423–1432. 10.1016/S1470-2045(20)30451-4 33035459

[B36] Greiner D. Bonaldi T. Eskeland R. Roemer E. Imhof A. (2005). Identification of a specific inhibitor of the histone methyltransferase SU(VAR)3-9. *Nat. Chem. Biol.* 1 143–145. 10.1038/nchembio721 16408017

[B37] Han X. Han Y. Zheng Y. Sun Q. Ma T. Zhang J. (2017). Chaetocin induces apoptosis in human melanoma cells through the generation of reactive oxygen species and the intrinsic mitochondrial pathway, and exerts its anti-tumor activity in vivo. *PLoS One* 12:e0175950. 10.1371/journal.pone.0175950 28419143 PMC5395229

[B38] Hauer M. H. Gasser S. M. (2017). Chromatin and nucleosome dynamics in DNA damage and repair. *Genes Dev.* 31 2204–2221. 10.1101/gad.307702.117 29284710 PMC5769766

[B39] He J. Chen X. Li B. Zhou W. Xiao J. He K. (2017). Chaetocin induces cell cycle arrest and apoptosis by regulating the ROS-mediated ASK-1/JNK signaling pathways. *Oncol. Rep.* 38 2489–2497. 10.3892/or.2017.5921 28849240

[B40] Higuchi F. Nagashima H. Ning J. Koerner M. V. A. Wakimoto H. Cahill D. P. (2020). Restoration of temozolomide sensitivity by PARP inhibitors in mismatch repair deficient glioblastoma is independent of base excision repair. *Clin. Cancer Res* 26 1690–1699. 10.1158/1078-0432.CCR-19-2000 31900275 PMC7192178

[B41] Hoeijmakers J. H. (2001). Genome maintenance mechanisms for preventing cancer. *Nature* 411 366–374. 10.1038/35077232 11357144

[B42] Hsiao K. Y. Mizzen C. A. (2013). Histone H4 deacetylation facilitates 53BP1 DNA damage signaling and double-strand break repair. *J. Mol. Cell Biol.* 5 157–165. 10.1093/jmcb/mjs066 23329852

[B43] Isham C. R. Tibodeau J. D. Jin W. Xu R. Timm M. M. Bible K. C. (2007). Chaetocin: a promising new antimyeloma agent with in vitro and in vivo activity mediated via imposition of oxidative stress. *Blood* 109 2579–2588. 10.1182/blood-2006-07-027326 17090648 PMC1852204

[B44] Italiano A. (2020). Targeting epigenetics in sarcomas through EZH2 inhibition. *J. Hematol. Oncol.* 13:33. 10.1186/s13045-020-00868-4 32264965 PMC7140314

[B45] Italiano A. Soria J. C. Toulmonde M. Michot J. M. Lucchesi C. Varga A. (2018). Tazemetostat, an EZH2 inhibitor, in relapsed or refractory B-cell non-Hodgkin lymphoma and advanced solid tumours: a first-in-human, open-label, phase 1 study. *Lancet Oncol.* 19 649–659. 10.1016/S1470-2045(18)30145-1 29650362

[B46] Iwasa E. Hamashima Y. Fujishiro S. Higuchi E. Ito A. Yoshida M. (2010). Total synthesis of (+)-chaetocin and its analogues: their histone methyltransferase G9a inhibitory activity. *J. Am. Chem. Soc.* 132 4078–4079. 10.1021/ja101280p 20210309

[B47] Jackson S. P. Bartek J. (2009). The DNA-damage response in human biology and disease. *Nature* 461 1071–1078. 10.1038/nature08467 19847258 PMC2906700

[B48] Jacquet K. Fradet-Turcotte A. Avvakumov N. Lambert J. P. Roques C. Pandita R. K. (2016). The TIP60 complex regulates bivalent chromatin recognition by 53BP1 through direct H4K20me binding and H2AK15 acetylation. *Mol. Cell.* 62 409–421. 10.1016/j.molcel.2016.03.031 27153538 PMC4887106

[B49] Karakashev S. Fukumoto T. Zhao B. Lin J. Wu S. Fatkhutdinov N. (2020). EZH2 inhibition sensitizes CARM1-high, homologous recombination proficient ovarian cancers to PARP inhibition. *Cancer Cell* 37 157.e6–167.e6. 10.1016/j.ccell.2019.12.015 32004442 PMC7155421

[B50] Kategaya L. Perumal S. K. Hager J. H. Belmont L. D. (2019). Werner syndrome helicase is required for the survival of cancer cells with microsatellite instability. *iScience* 13 488–497. 10.1016/j.isci.2019.02.006 30898619 PMC6441948

[B51] Kouzarides T. (2007). Chromatin modifications and their function. *Cell* 128 693–705. 10.1016/j.cell.2007.02.005 17320507

[B52] Kulis M. Esteller M. (2010). DNA methylation and cancer. *Adv. Genet.* 70 27–56. 10.1016/B978-0-12-380866-0.60002-2 20920744

[B53] Lai Y. S. Chen J. Y. Tsai H. J. Chen T. Y. Hung W. C. (2015). The SUV39H1 inhibitor chaetocin induces differentiation and shows synergistic cytotoxicity with other epigenetic drugs in acute myeloid leukemia cells. *Blood Cancer J.* 5:e313. 10.1038/bcj.2015.37 25978433 PMC4476016

[B54] Lavin M. F. Kozlov S. Gatei M. Kijas A. W. (2015). ATM-dependent phosphorylation of all three members of the mrn complex: from sensor to adaptor. *Biomolecules* 5 2877–2902. 10.3390/biom5042877 26512707 PMC4693261

[B55] Lee H. J. Yoon C. Schmidt B. Park D. J. Zhang A. Y. Erkizan H. V. (2013). Combining PARP-1 inhibition and radiation in Ewing sarcoma results in lethal DNA damage. *Mol. Cancer Ther.* 12 2591–2600. 10.1158/1535-7163.MCT-13-0338 23966622 PMC3823674

[B56] Leichman L. Groshen S. O’Neil B. H. Messersmith W. Berlin J. Chan E. (2016). Phase II study of olaparib (AZD-2281) after standard systemic therapies for disseminated colorectal cancer. *Oncologist* 21 172–177. 10.1634/theoncologist.2015-0319 26786262 PMC4746089

[B57] Lesueur P. Lequesne J. Grellard J. M. Dugue A. Coquan E. Brachet P. E. (2019). Phase I/IIa study of concomitant radiotherapy with olaparib and temozolomide in unresectable or partially resectable glioblastoma: OLA-TMZ-RTE-01 trial protocol. *BMC Cancer* 19:198. 10.1186/s12885-019-5413-y 30832617 PMC6399862

[B58] Li L. Wang Y. (2017). Cross-talk between the H3K36me3 and H4K16ac histone epigenetic marks in DNA double-strand break repair. *J. Biol. Chem.* 292 11951–11959. 10.1074/jbc.M117.788224 28546430 PMC5512086

[B59] Li Z. Bao J. Qi Y. Zhang J. Z. H. (2020). Computational approaches to studying methylated H4K20 recognition by DNA repair factor 53BP1. *Phys. Chem. Chem. Phys.* 22 6136–6144. 10.1039/c9cp05635a 32124883

[B60] Li Z. Huang L. Wei L. Hou Z. Ye W. Huang S. (2019). Chaetocin induces caspase-dependent apoptosis in ovarian cancer cells via the generation of reactive oxygen species. *Oncol. Lett.* 18 1915–1921. 10.3892/ol.2019.10507 31423261 PMC6614685

[B61] Liao X. Fan Y. Hou J. Chen X. Xu X. Yang Y. (2019). Identification of Chaetocin as a Potent non-ROS-mediated anticancer drug candidate for gastric Cancer. *J. Cancer* 10 3678–3690. 10.7150/jca.32803 31333785 PMC6636309

[B62] Lieb S. Blaha-Ostermann S. Kamper E. Rippka J. Schwarz C. Ehrenhofer-Wolfer K. (2019). Werner syndrome helicase is a selective vulnerability of microsatellite instability-high tumor cells. *eLife* 8:e43333. 10.7554/eLife.43333 30910006 PMC6435321

[B63] Liu J. Luo S. Zhao H. Liao J. Li J. Yang C. (2012). Structural mechanism of the phosphorylation-dependent dimerization of the MDC1 forkhead-associated domain. *Nucleic Acids Res.* 40 3898–3912. 10.1093/nar/gkr1296 22234877 PMC3351156

[B64] Liu X. Guo S. Liu X. Su L. (2015). Chaetocin induces endoplasmic reticulum stress response and leads to death receptor 5-dependent apoptosis in human non-small cell lung cancer cells. *Apoptosis* 20 1499–1507. 10.1007/s10495-015-1167-4 26349783

[B65] Lottersberger F. Bothmer A. Robbiani D. F. Nussenzweig M. C. de Lange T. (2013). Role of 53BP1 oligomerization in regulating double-strand break repair. *Proc. Natl. Acad. Sci. U.S.A.* 110 2146–2151. 10.1073/pnas.1222617110 23345425 PMC3568336

[B66] Lou Z. Minter-Dykhouse K. Franco S. Gostissa M. Rivera M. A. Celeste A. (2006). MDC1 maintains genomic stability by participating in the amplification of ATM-dependent DNA damage signals. *Mol. Cell.* 21 187–200. 10.1016/j.molcel.2005.11.025 16427009

[B67] Maurel J. Lopez-Pousa A. de Las Penas R. Fra J. Martin J. Cruz J. (2009). Efficacy of sequential high-dose doxorubicin and ifosfamide compared with standard-dose doxorubicin in patients with advanced soft tissue sarcoma: an open-label randomized phase II study of the Spanish group for research on sarcomas. *J. Clin. Oncol.* 27 1893–1898. 19273704 10.1200/JCO.2008.19.2930

[B68] McMahon M. Frangova T. G. Henderson C. J. Wolf C. R. (2016). Olaparib, monotherapy or with ionizing radiation, exacerbates DNA damage in normal tissues: insights from a new p21 reporter mouse. *Mol. Cancer Res.* 14 1195–1203. 10.1158/1541-7786.MCR-16-0108 27604276 PMC5136472

[B69] Mirman Z. de Lange T. (2020). 53BP1: a DSB escort. *Genes Dev.* 34 7–23. 10.1101/gad.333237.119 31896689 PMC6938671

[B70] Monsalve D. M. Campillo-Marcos I. Salzano M. Sanz-Garcia M. Cantarero L. Lazo P. A. (2016). VRK1 phosphorylates and protects NBS1 from ubiquitination and proteasomal degradation in response to DNA damage. *Biochim. Biophys. Acta Mol. Cell Res.* 1863 760–769. 10.1016/j.bbamcr.2016.02.005 26869104

[B71] Moura D. S. Campillo-Marcos I. Vazquez-Cedeira M. Lazo P. A. (2018). VRK1 and AURKB form a complex that cross inhibit their kinase activity and the phosphorylation of histone H3 in the progression of mitosis. *Cell Mol. Life Sci.* 76 2591–2611. 10.1007/s00018-018-2746-7 29340707 PMC6003988

[B72] Murr R. Loizou J. I. Yang Y. G. Cuenin C. Li H. Wang Z. Q. (2006). Histone acetylation by Trrap-Tip60 modulates loading of repair proteins and repair of DNA double-strand breaks. *Nat. Cell. Biol.* 8 91–99. 10.1038/ncb1343 16341205

[B73] Navarro-Carrasco E. Lazo P. A. (2021). VRK1 depletion facilitates the synthetic lethality of temozolomide and olaparib in glioblastoma cells. *Front. Cell Dev. Biol.* 9:683038. 10.3389/fcell.2021.683038 34195200 PMC8237761

[B74] Ozyerli-Goknar E. Sur-Erdem I. Seker F. Cingoz A. Kayabolen A. Kahya-Yesil Z. (2019). The fungal metabolite chaetocin is a sensitizer for pro-apoptotic therapies in glioblastoma. *Cell Death Dis.* 10:894. 10.1038/s41419-019-2107-y 31772153 PMC6879621

[B75] Panier S. Boulton S. J. (2014). Double-strand break repair: 53BP1 comes into focus. *Nat. Rev. Mol. Cell Biol.* 15 7–18. 10.1038/nrm3719 24326623

[B76] Pei D. Zhang Y. Zheng J. (2012). Regulation of p53: a collaboration between Mdm2 and Mdmx. *Oncotarget* 3 228–235. 10.18632/oncotarget.443 22410433 PMC3359881

[B77] Pei H. Zhang L. Luo K. Qin Y. Chesi M. Fei F. (2011). MMSET regulates histone H4K20 methylation and 53BP1 accumulation at DNA damage sites. *Nature* 470 124–128. 10.1038/nature09658 21293379 PMC3064261

[B78] Pollard D. A. Pollard T. D. Pollard K. S. (2019). Empowering statistical methods for cellular and molecular biologists. *Mol. Biol. Cell* 30 1359–1368. 10.1091/mbc.E15-02-0076 31145670 PMC6724699

[B79] Polo S. E. (2015). Reshaping chromatin after DNA damage: the choreography of histone proteins. *J. Mol. Biol.* 427 626–636. 10.1016/j.jmb.2014.05.025 24887097 PMC5111727

[B80] Ramakrishnan Geethakumari P. Schiewer M. J. Knudsen K. E. Kelly W. K. (2017). PARP inhibitors in prostate cancer. *Curr. Treat. Options. Oncol.* 18:37. 10.1007/s11864-017-0480-2 28540598

[B81] Robson M. Im S. A. Senkus E. Xu B. Domchek S. M. Masuda N. (2017). Olaparib for metastatic breast cancer in patients with a germline BRCA mutation. *N. Engl. J. Med.* 377 523–533. 10.1056/NEJMoa1706450 28578601

[B82] Rothbart S. B. Baylin S. B. (2020). Epigenetic therapy for epithelioid sarcoma. *Cell* 181:211. 10.1016/j.cell.2020.03.042 32302562

[B83] Salzano M. Sanz-Garcia M. Monsalve D. M. Moura D. S. Lazo P. A. (2015). VRK1 chromatin kinase phosphorylates H2AX and is required for foci formation induced by DNA damage. *Epigenetics* 10 373–383. 10.1080/15592294.2015.1028708 25923214 PMC4623420

[B84] Salzano M. Vazquez-Cedeira M. Sanz-Garcia M. Valbuena A. Blanco S. Fernandez I. F. (2014). Vaccinia-related kinase 1 (VRK1) confers resistance to DNA-damaging agents in human breast cancer by affecting DNA damage response. *Oncotarget* 5 1770–1778. 10.18632/oncotarget.1678 24731990 PMC4039124

[B85] Sanz-Garcia M. Monsalve D. M. Sevilla A. Lazo P. A. (2012). Vaccinia-related Kinase 1 (VRK1) is an upstream nucleosomal kinase required for the assembly of 53BP1 foci in response to ionizing radiation-induced DNA damage. *J. Biol. Chem.* 287 23757–23768. 10.1074/jbc.M112.353102 22621922 PMC3390650

[B86] Shechter D. Dormann H. L. Allis C. D. Hake S. B. (2007). Extraction, purification and analysis of histones. *Nat. Protoc.* 2 1445–1457. 10.1038/nprot.2007.202 17545981

[B87] Shibata A. (2017). Regulation of repair pathway choice at two-ended DNA double-strand breaks. *Mutat. Res.* 803-805 51–55. 10.1016/j.mrfmmm.2017.07.011 28781144

[B88] Shilatifard A. (2006). Chromatin modifications by methylation and ubiquitination: implications in the regulation of gene expression. *Annu. Rev. Biochem.* 75 243–269. 10.1146/annurev.biochem.75.103004.142422 16756492

[B89] Soldani C. Scovassi A. I. (2002). Poly(ADP-ribose) polymerase-1 cleavage during apoptosis: an update. *Apoptosis* 7 321–328. 10.1023/a:1016119328968 12101391

[B90] Soumyanarayanan U. Dymock B. W. (2016). Recently discovered EZH2 and EHMT2 (G9a) inhibitors. *Future Med. Chem.* 8 1635–1654. 10.4155/fmc-2016-0096 27548656

[B91] Srinivasan G. Sidhu G. S. Williamson E. A. Jaiswal A. S. Najmunnisa N. Wilcoxen K. (2017). Synthetic lethality in malignant pleural mesothelioma with PARP1 inhibition. *Cancer Chemother. Pharmacol.* 80 861–867. 10.1007/s00280-017-3401-y 28756516 PMC5608777

[B92] Tang J. Cho N. W. Cui G. Manion E. M. Shanbhag N. M. Botuyan M. V. (2013). Acetylation limits 53BP1 association with damaged chromatin to promote homologous recombination. *Nat. Struct. Mol. Biol.* 20 317–325. 10.1038/nsmb.2499 23377543 PMC3594358

[B93] Tewari K. S. Eskander R. N. Monk B. J. (2015). Development of olaparib for BRCA-deficient recurrent epithelial ovarian cancer. *Clin. Cancer Res.* 21 3829–3835. 10.1158/1078-0432.CCR-15-0088 26169965

[B94] Tibodeau J. D. Benson L. M. Isham C. R. Owen W. G. Bible K. C. (2009). The anticancer agent chaetocin is a competitive substrate and inhibitor of thioredoxin reductase. *Antioxid. Redox. Signal.* 11 1097–1106. 10.1089/ARS.2008.2318 18999987 PMC2842135

[B95] Toh T. B. Lim J. J. Chow E. K. (2017). Epigenetics in cancer stem cells. *Mol. Cancer* 16:29. 10.1186/s12943-017-0596-9 28148257 PMC5286794

[B96] van Attikum H. Gasser S. M. (2005). The histone code at DNA breaks: a guide to repair?. *Nat. Rev. Mol. Cell Biol.* 6 757–765. 16167054 10.1038/nrm1737

[B97] van Gent D. C. Hoeijmakers J. H. Kanaar R. (2001). Chromosomal stability and the DNA double-stranded break connection. *Nat. Rev. Genet.* 2 196–206. 10.1038/35056049 11256071

[B98] Wakeman T. P. Wang Q. Feng J. Wang X. F. (2012). Bat3 facilitates H3K79 dimethylation by DOT1L and promotes DNA damage-induced 53BP1 foci at G1/G2 cell-cycle phases. *EMBO J.* 31 2169–2181. 10.1038/emboj.2012.50 22373577 PMC3343460

[B99] Wang Z. Patel D. J. (2013). Small molecule epigenetic inhibitors targeted to histone lysine methyltransferases and demethylases. *Q. Rev. Biophys.* 46 349–373. 10.1017/S0033583513000085 23991894 PMC4696758

[B100] Wen C. Wang H. Wu X. He L. Zhou Q. Wang F. (2019). ROS-mediated inactivation of the PI3K/AKT pathway is involved in the antigastric cancer effects of thioredoxin reductase-1 inhibitor chaetocin. *Cell Death Dis.* 10:809. 10.1038/s41419-019-2035-x 31649256 PMC6813365

[B101] Wheeler D. A. Takebe N. Hinoue T. Hoadley K. A. Cardenas M. F. Hamilton A. M. (2021). Molecular features of cancers exhibiting exceptional responses to treatment. *Cancer Cell* 39 1–16. 10.1016/j.ccell.2020.10.015 33217343 PMC8478080

[B102] Wilson M. D. Benlekbir S. Fradet-Turcotte A. Sherker A. Julien J. P. McEwan A. (2016). The structural basis of modified nucleosome recognition by 53BP1. *Nature* 536 100–103. 10.1038/nature18951 27462807

[B103] Zhao B. Rothenberg E. Ramsden D. A. Lieber M. R. (2020). The molecular basis and disease relevance of non-homologous DNA end joining. *Nat. Rev. Mol. Cell Biol.* 21 765–781. 10.1038/s41580-020-00297-8 33077885 PMC8063501

