# Supplementary material for: Lysine Methyltransferase Inhibitors Impair H4K20me2 and 53BP1 Foci in Response to DNA Damage in Sarcomas, a Synthetic Lethality Strategy
Source: Front Cell Dev Biol. 2021 Sep 3;9:715126. doi: 10.3389/fcell.2021.715126 (PMC8446283; doi:10.3389/fcell.2021.715126)
Supplement: Supplementary file 1 [file Data_Sheet_1.PDF]

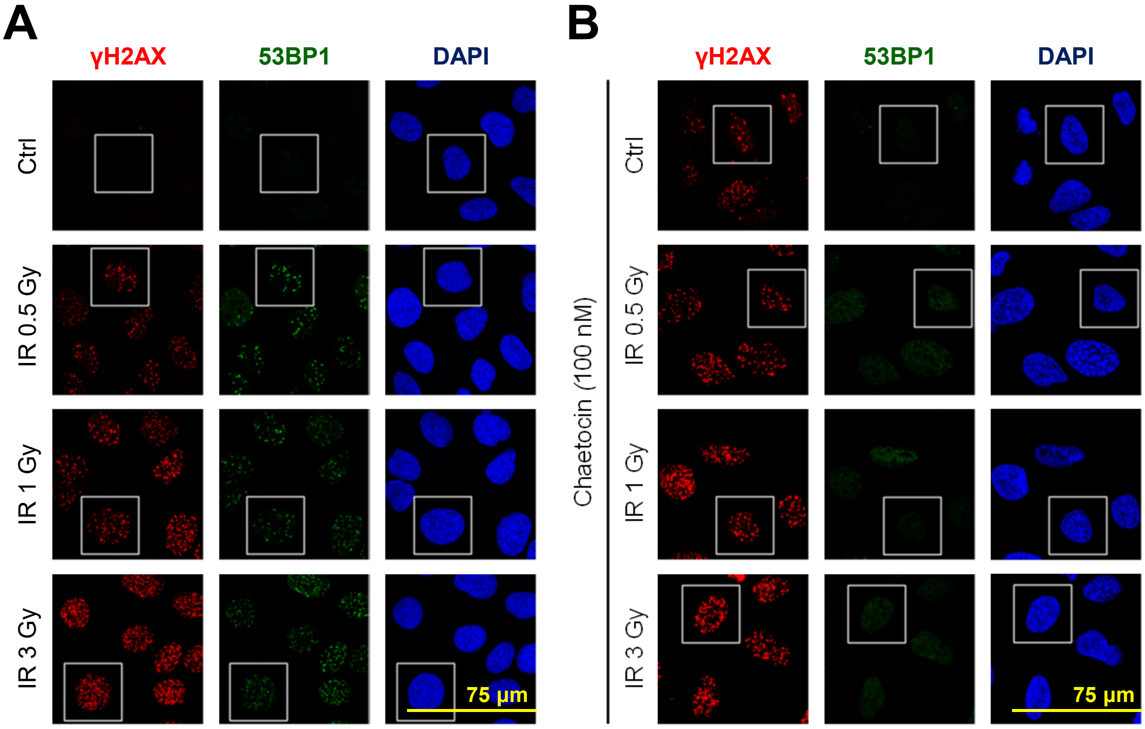

Supplementary Figure 1. Chaetocin impairs 53BP1 foci formation induced by IR in U2OS osteosarcoma cells deprived of serum. **A**. Assembly of γH2AX and 53BP1 foci in response to different doses of IR. **B**. Effect of chaetocin on γH2AX and 53BP1 foci formation after inducing DNA damage by IR. The detail images selected for Figure 1 are indicated by boxes. Ctrl: control without IR.
