# Supplementary material for: Lysine Methyltransferase Inhibitors Impair H4K20me2 and 53BP1 Foci in Response to DNA Damage in Sarcomas, a Synthetic Lethality Strategy
Source: Front Cell Dev Biol. 2021 Sep 3;9:715126. doi: 10.3389/fcell.2021.715126 (PMC8446283; doi:10.3389/fcell.2021.715126)
Supplement: Supplementary file 2 [file Data_Sheet_2.PDF]

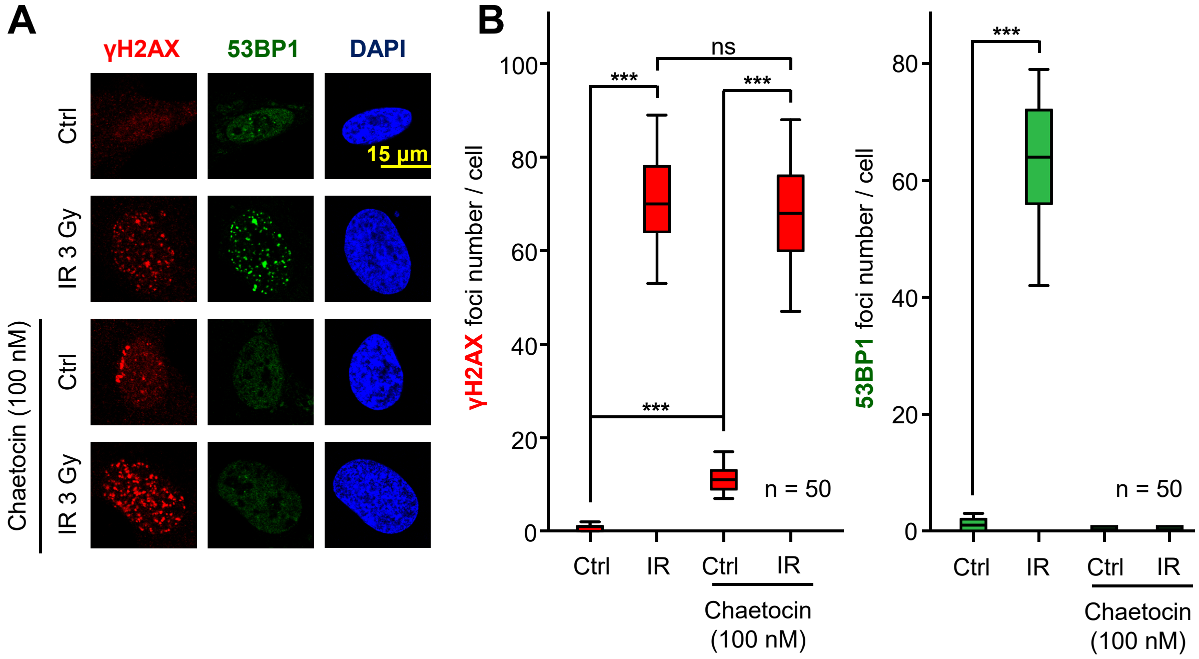

Supplementary Figure 2. Chaetocin impairs the assembly of 53BP1 foci induced by IR in SK-LMS-1 leiomyosarcoma serum-deprived cells. **A**. Effect of chaetocin on γH2AX and 53BP1 foci formation after inducing DNA damage with different doses of IR. **B**. Quantification of γH2AX (left) and 53BP1 (right) foci in response to chaetocin and/or IR. Ctrl: control without IR. ns: not significant, *** $p < 0.001$.
