# Supplementary material for: Lysine Methyltransferase Inhibitors Impair H4K20me2 and 53BP1 Foci in Response to DNA Damage in Sarcomas, a Synthetic Lethality Strategy
Source: Front Cell Dev Biol. 2021 Sep 3;9:715126. doi: 10.3389/fcell.2021.715126 (PMC8446283; doi:10.3389/fcell.2021.715126)
Supplement: Supplementary file 3 [file Data_Sheet_3.PDF]

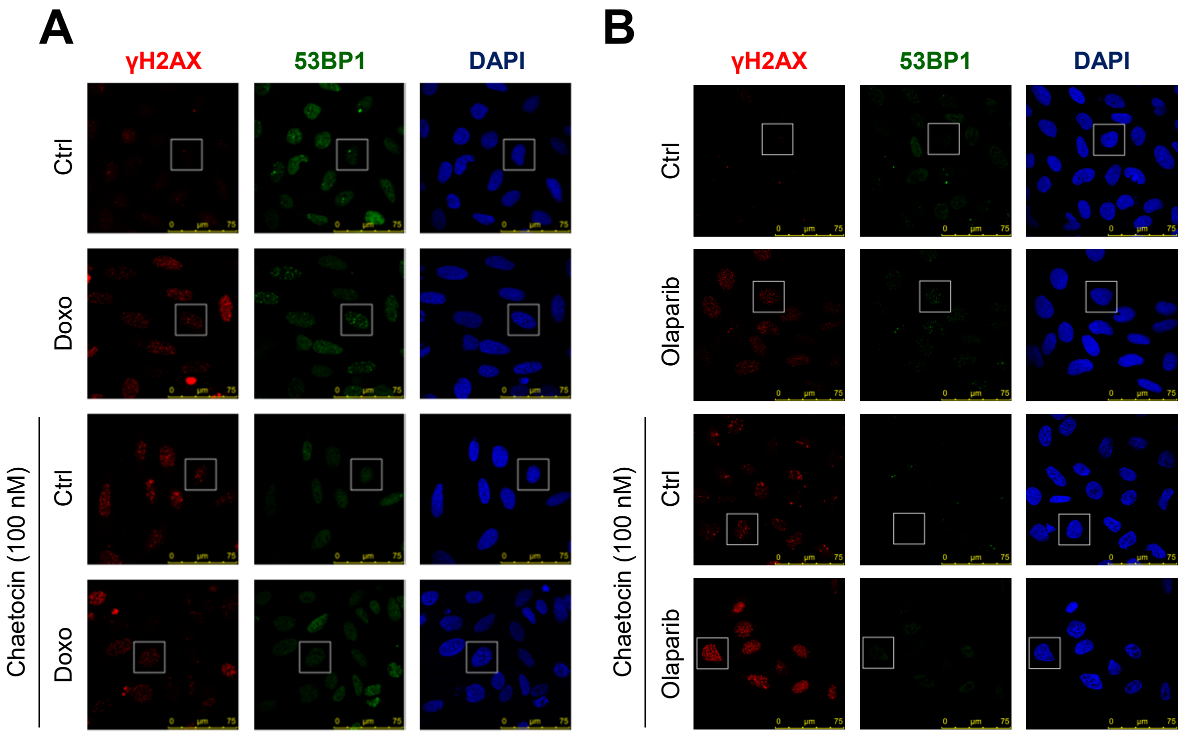

Supplementary Figure 3. Effect of chaetocin on the formation of γH2AX and 53BP1 foci after inducing DSBs with doxorubicin (Doxo) (**A**) and olaparib (Olap) (**B**) in U2OS cells deprived of serum mitogenic signals. The detail images selected for Figure 2 are indicated by boxes. Ctrl: control without doxorubicin or olaparib, as appropiate.
