# Supplementary material for: Lysine Methyltransferase Inhibitors Impair H4K20me2 and 53BP1 Foci in Response to DNA Damage in Sarcomas, a Synthetic Lethality Strategy
Source: Front Cell Dev Biol. 2021 Sep 3;9:715126. doi: 10.3389/fcell.2021.715126 (PMC8446283; doi:10.3389/fcell.2021.715126)
Supplement: Supplementary file 4 [file Data_Sheet_4.PDF]

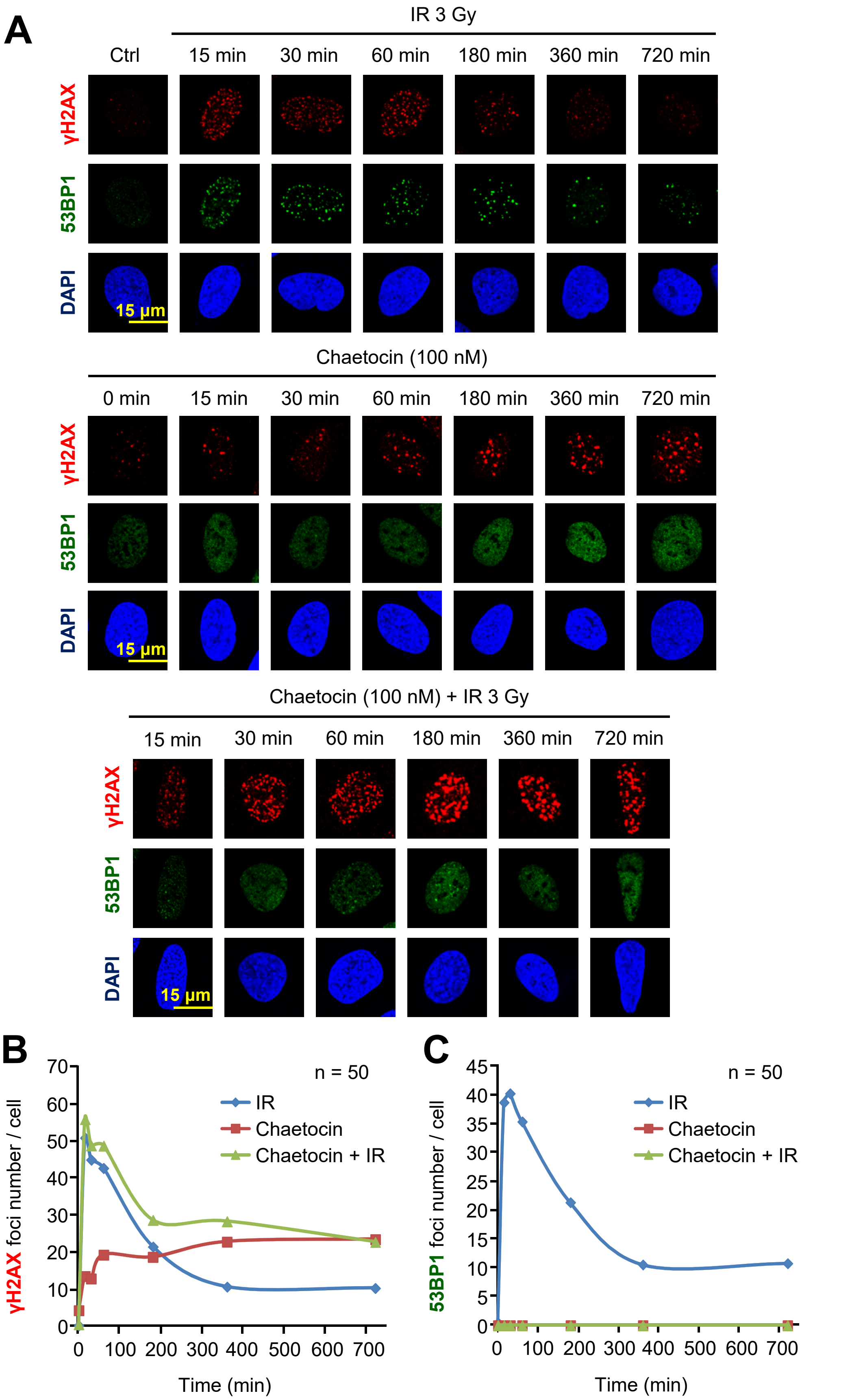

Supplementary Figure 4. The impairment of 53BP1 foci formation induced by chaetocin is independent of the post-irradiation time in U2OS cells. **A**. Assembly of γH2AX and 53BP1 foci in response to IR (top), chaetocin (center) and their combination (bottom). **B**.**C**. Quantification of γH2AX (B) and 53BP1(C) foci after chaetocin and/or IR treatments. Ctrl: control.
