# Supplementary material for: Lysine Methyltransferase Inhibitors Impair H4K20me2 and 53BP1 Foci in Response to DNA Damage in Sarcomas, a Synthetic Lethality Strategy
Source: Front Cell Dev Biol. 2021 Sep 3;9:715126. doi: 10.3389/fcell.2021.715126 (PMC8446283; doi:10.3389/fcell.2021.715126)
Supplement: Supplementary file 5 [file Data_Sheet_5.PDF]

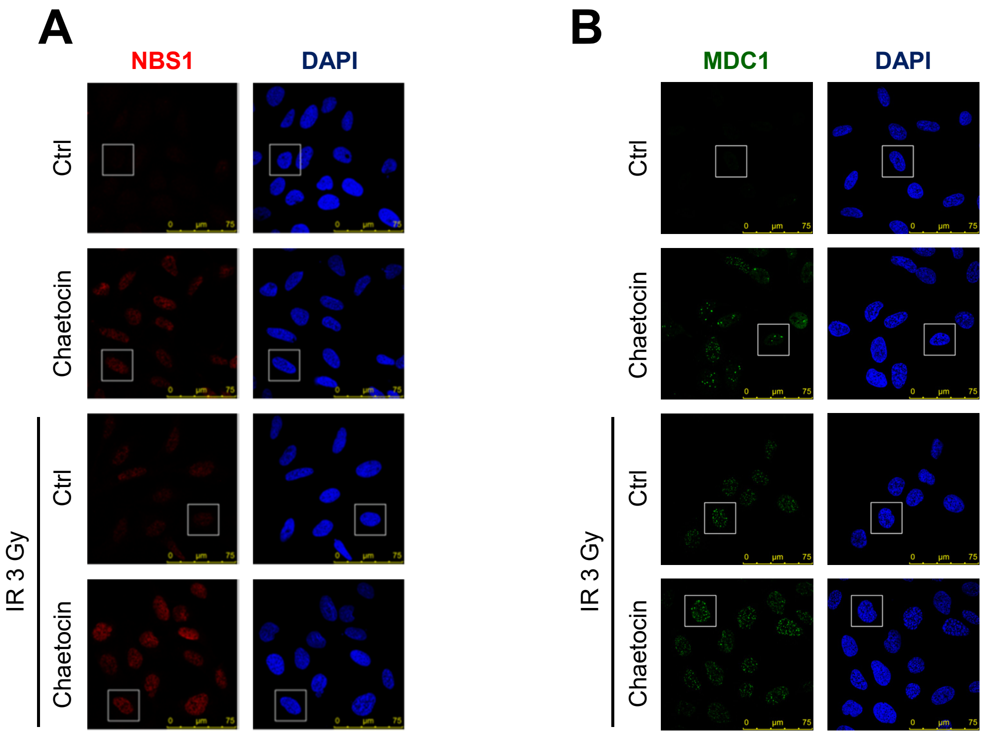

Supplementary Figure 5. Chaetocin does not interfere with MDC1 foci formation and NBS1 accumulation after inducing DSBs by IR in U2OS cells deprived of serum. **A**. Effect of chaetocin on MDC1 foci in response to IR (3 Gy). **B**. Effect of chaetocin on NBS1 accumulation in response to IR (3 Gy) in U2OS cells. The detail images selected for Figure 3 are indicated by boxes. Ctrl: control without chaetocin.
