# Supplementary material for: Lysine Methyltransferase Inhibitors Impair H4K20me2 and 53BP1 Foci in Response to DNA Damage in Sarcomas, a Synthetic Lethality Strategy
Source: Front Cell Dev Biol. 2021 Sep 3;9:715126. doi: 10.3389/fcell.2021.715126 (PMC8446283; doi:10.3389/fcell.2021.715126)
Supplement: Supplementary file 6 [file Data_Sheet_6.PDF]

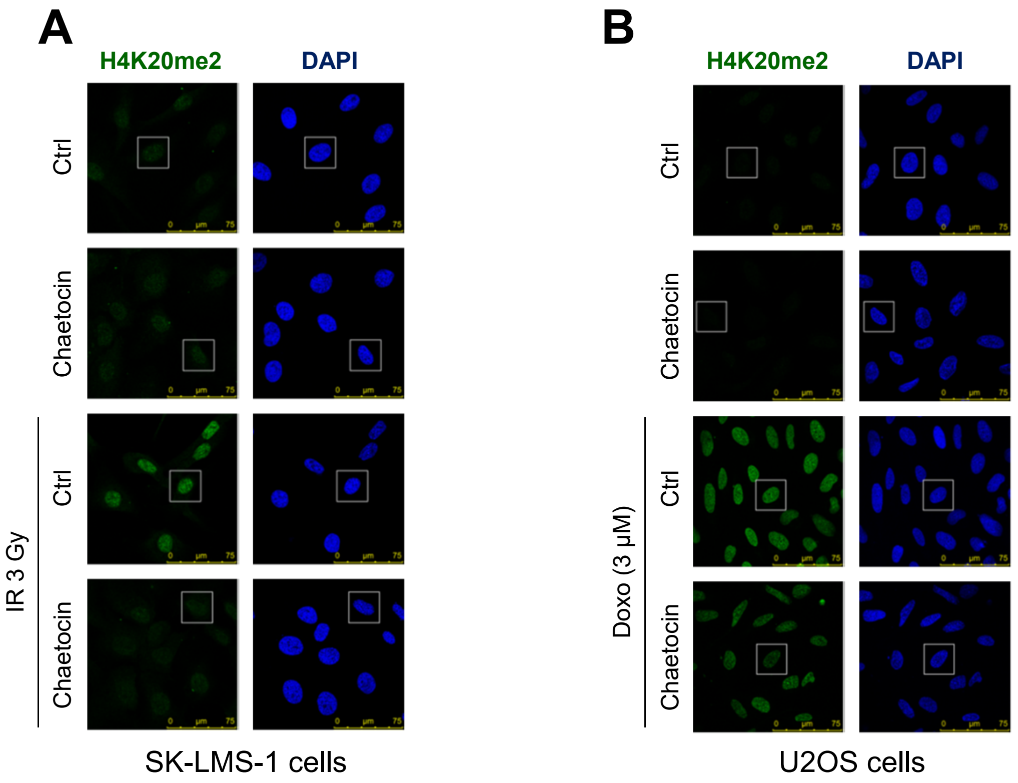

Supplementary Figure 6. Effect of chaetocin on H4K20me2 induced by IR or doxorubicin. **A.** Effect of chaetocin on H4K20me2 induced by IR in SK-LMS-1 cells. **B.** Effect of chaetocin on H4K20me2 induced by doxorubicin in U2OS cells. The detail images selected for Figure 4 are indicated by boxes. Ctrl: control without chaetocin.
