# Supplementary material for: Lysine Methyltransferase Inhibitors Impair H4K20me2 and 53BP1 Foci in Response to DNA Damage in Sarcomas, a Synthetic Lethality Strategy
Source: Front Cell Dev Biol. 2021 Sep 3;9:715126. doi: 10.3389/fcell.2021.715126 (PMC8446283; doi:10.3389/fcell.2021.715126)
Supplement: Supplementary file 7 [file Data_Sheet_7.PDF]

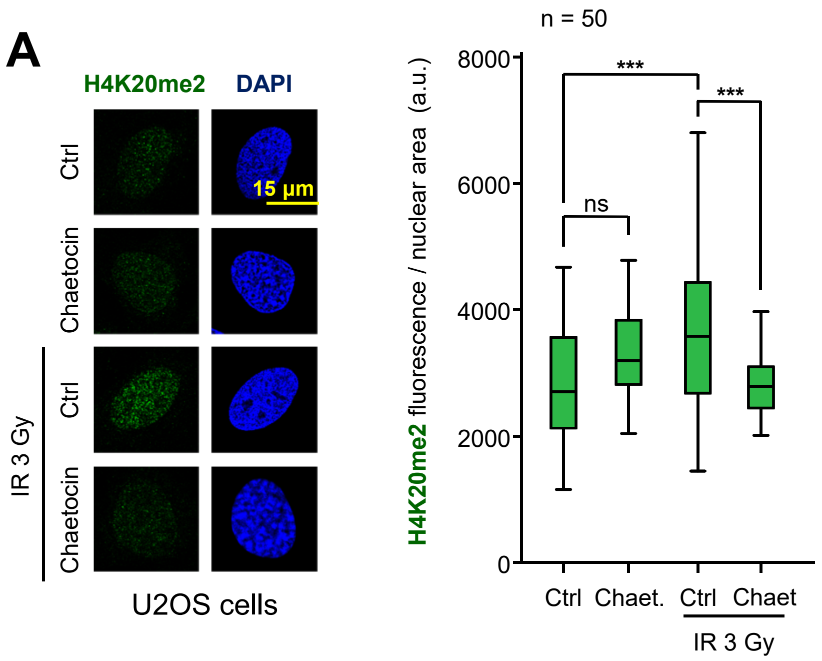

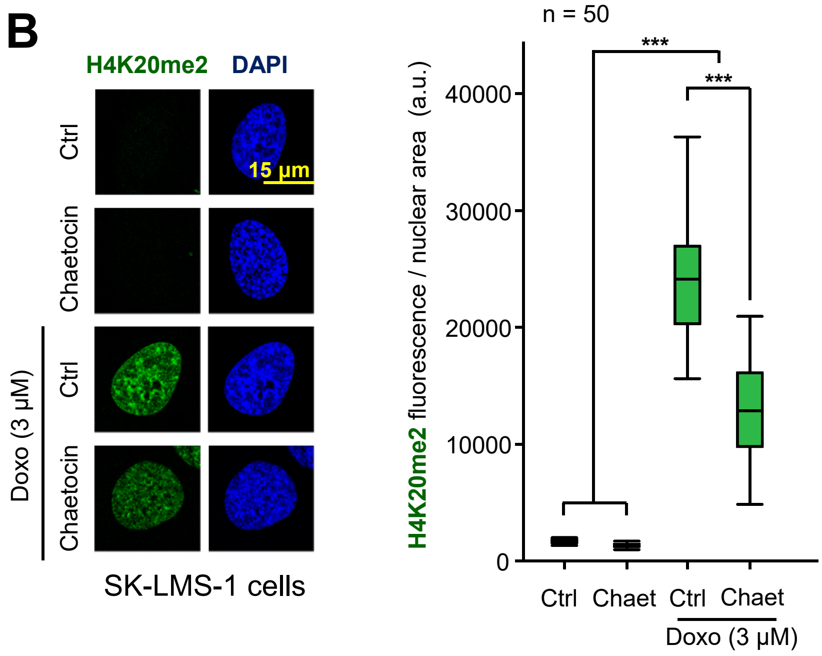

Supplementary Figure 7. Effect of chaetocin on H4K20me2 induced by IR or doxorubicin. **A.** Effect of chaetocin on H4K20me2 induced by IR in U2OS cells. **B.** Effect of chaetocin on H4K20me2 induced by doxorubicin in SK-LMS-1 cells. Quantification of nuclear fluorescence associated with H4K20 demethylation in response to chaetocin, IR, doxorubicin or their combination (right). ns: not significant, *** $p < 0.001$. Ctrl: control without chaetocin. Chaet: chaetocin (100 nM).
