# Supplementary material for: Lysine Methyltransferase Inhibitors Impair H4K20me2 and 53BP1 Foci in Response to DNA Damage in Sarcomas, a Synthetic Lethality Strategy
Source: Front Cell Dev Biol. 2021 Sep 3;9:715126. doi: 10.3389/fcell.2021.715126 (PMC8446283; doi:10.3389/fcell.2021.715126)
Supplement: Supplementary file 8 [file Data_Sheet_8.PDF]

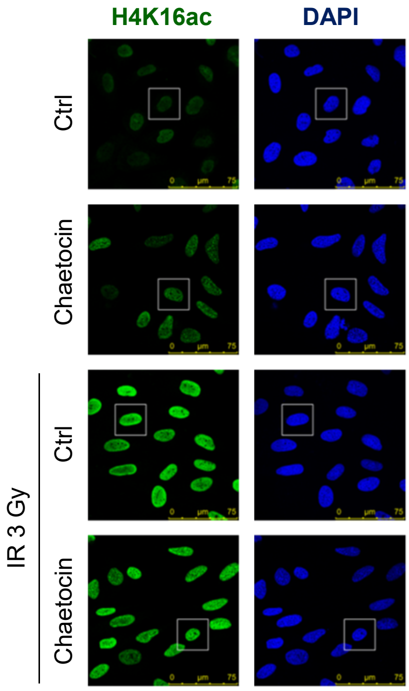

Supplementary Figure 8. Effect of chaetocin on the acetylation of histone H4 in lysine 16 (H4K16ac) in U2OS cells treated with IR. The detail images selected for Figure 5 are indicated by boxes. Ctrl: control without chaetocin.
