# Supplementary material for: Lysine Methyltransferase Inhibitors Impair H4K20me2 and 53BP1 Foci in Response to DNA Damage in Sarcomas, a Synthetic Lethality Strategy
Source: Front Cell Dev Biol. 2021 Sep 3;9:715126. doi: 10.3389/fcell.2021.715126 (PMC8446283; doi:10.3389/fcell.2021.715126)
Supplement: Supplementary file 9 [file Data_Sheet_9.PDF]

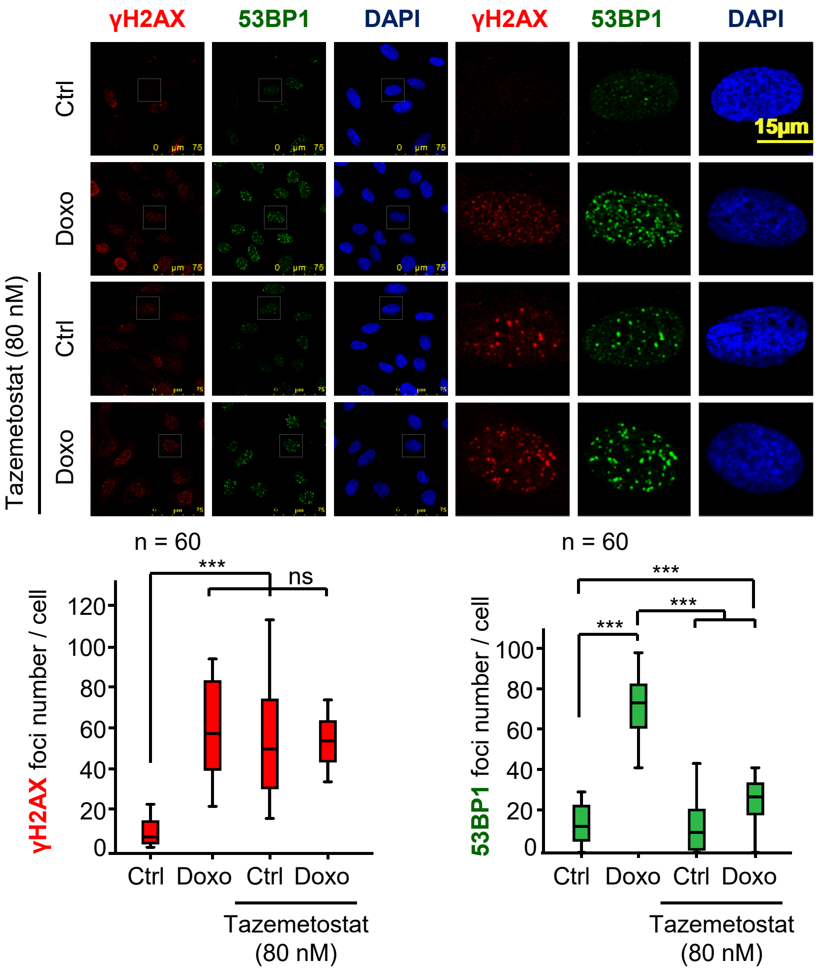

Supplementary Figure 9. Effect of tazemetostat on γH2AX and 53BP1 foci in response to doxorubicin treatment in SK-LMS-1 leiomyosarcoma cells deprived of serum. Effect of tazemetostat on the formation of γH2AX (red) and 53BP1 (green) foci. The detail images selected indicated by boxes are shown to the right. Graphs at the bottom show the quantification of γH2AX and 53BP1 foci. ns: not significant. *** $p < 0.001$. Ctrl: control without doxorubicin.
