# Supplementary material for: Lysine Methyltransferase Inhibitors Impair H4K20me2 and 53BP1 Foci in Response to DNA Damage in Sarcomas, a Synthetic Lethality Strategy
Source: Front Cell Dev Biol. 2021 Sep 3;9:715126. doi: 10.3389/fcell.2021.715126 (PMC8446283; doi:10.3389/fcell.2021.715126)
Supplement: Supplementary file 10 [file Data_Sheet_10.PDF]

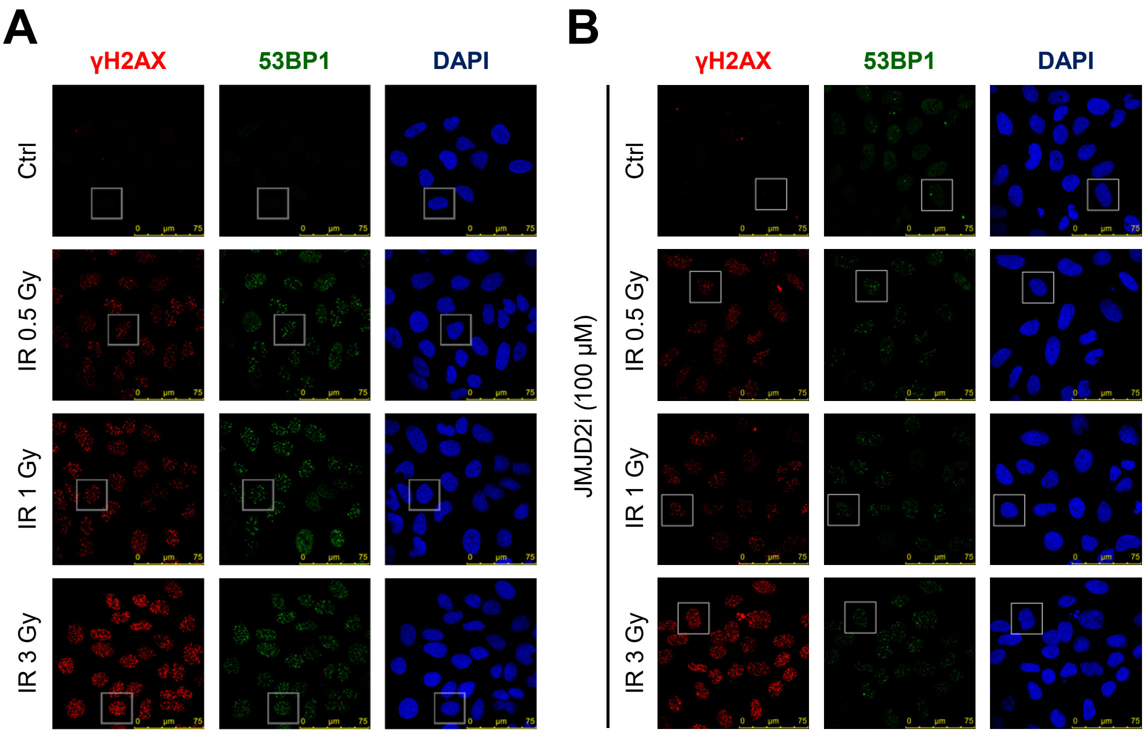

Supplementary Figure 10. The assembly of 53BP1 foci is not affected by JMJD2i treatment, which inhibits lysine demethylation, after inducing DSBs with IR in U2OS cells. **A**. Assembly of γH2AX and 53BP1 foci in response to different doses of IR. **B**. Effect of JMJD2i on γH2AX and 53BP1 foci formation after inducing DNA damage by IR. The detail images selected for Figure 8 are indicated by boxes. Ctrl: control without IR.
