# Supplementary material for: Lysine Methyltransferase Inhibitors Impair H4K20me2 and 53BP1 Foci in Response to DNA Damage in Sarcomas, a Synthetic Lethality Strategy
Source: Front Cell Dev Biol. 2021 Sep 3;9:715126. doi: 10.3389/fcell.2021.715126 (PMC8446283; doi:10.3389/fcell.2021.715126)
Supplement: Supplementary file 11 [file Data_Sheet_11.PDF]

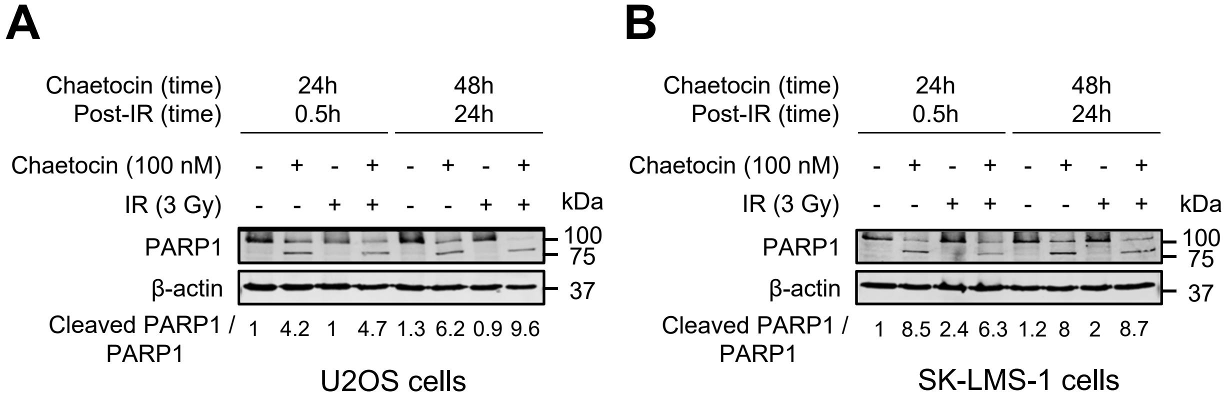

Supplementary Figure 11. Chaetocin induces apoptotic PARP1 cleavage in response to DNA damage caused by IR in sarcoma cells. **A**. Effect of chaetocin and/or IR on PARP1 cleavage at different points in time in U2OS osteosarcoma cells deprived of serum mitogenic signals. **B.** Effect of chaetocin and/or IR on PARP1 cleavage at different time points in SK-LMS-1 leiomyosarcoma cells.
