# Supplementary material for: Lysine Methyltransferase Inhibitors Impair H4K20me2 and 53BP1 Foci in Response to DNA Damage in Sarcomas, a Synthetic Lethality Strategy
Source: Front Cell Dev Biol. 2021 Sep 3;9:715126. doi: 10.3389/fcell.2021.715126 (PMC8446283; doi:10.3389/fcell.2021.715126)
Supplement: Supplementary file 12 [file Table_1.pdf]

Supplementary Table 1

Reagents used in this work

Reagent	Reference	Supplier
Doxorubicin	16416646	Fisher BioReagents
Chaetocin	C9492	Sigma-Aldrich-Merck
Olaparib	O-9201	LC Laboratories
Tazemetostat	S7128	Selleck
JMJD2i	420201	Calbiochem (Millipore)
NaF	20154	Sigma-Aldrich-Merck
Hepes	H3375	Sigma-Aldrich-Merck
Triton X-100	T8787	Sigma-Aldrich-Merck
SDS	L3771	Sigma-Aldrich-Merck
Na orthovanadate	450243	Sigma-Aldrich-Merck
PMSF	78830	Sigma-Aldrich-Merck
Aprotinin	A6103	Sigma-Aldrich-Merck
Leupeptin	L9783	Sigma-Aldrich-Merck
Tween 20	P2287	Sigma-Aldrich-Merck
